# Differential epithelial and stromal *LGR5* expression in ovarian carcinogenesis

**DOI:** 10.1038/s41598-022-15234-2

**Published:** 2022-07-01

**Authors:** Hyesung Kim, Dong Hui Lee, Eunsun Park, Jae Kyung Myung, Jeong Hwan Park, Dong Il Kim, Se Ik Kim, Maria Lee, Younghoon Kim, Chul Min Park, Chang Lim Hyun, Young Hee Maeng, Cheol Lee, Bogun Jang

**Affiliations:** 1 Department of Pathology, Jeju National University School of Medicine, 15, Aran-13 gil, Jeju-si, Jeju-do 63241 South Korea; 2 Department of Pathology, Hanyang University College of Medicine, Seoul, South Korea; 3 Department of Pathology, SMG-SNU Boramae Medical Center, Seoul, South Korea; 4 Department of Pathology, Green Cross Laboratories, Yongin, Gyeonggi South Korea; 5 Department of Obstetrics and Gynecology, Seoul National University College of Medicine, Seoul, South Korea; 6 Laboratory of Epigenetics, Cancer Research Institute, Seoul National University College of Medicine, Seoul, South Korea; 7 Department of Obstetrics and Gynecology, Jeju National University School of Medicine, Jeju, South Korea; 8 Department of Pathology, Seoul National University College of Medicine, 101 Daehak-ro, Jongno-gu, Seoul, 03080 South Korea

**Keywords:** Tumour biomarkers, Ovarian cancer

## Abstract

Lgr5 has been identified as a marker of the stem/progenitor cells in the murine ovary and oviduct by lineage tracing. However, little is known regarding *LGR5* expression or its functional significance in human ovary tissues. Here, using RNA in situ hybridization and/or immunohistochemistry, we thoroughly investigated *LGR5* expression in normal human ovaries, fallopian tubes and various ovarian tumors. We discovered that *LGR5* expression is negligible in the human ovary surface epithelium, whereas ovarian stromal cells normally express low levels of *LGR5*. Remarkably, fallopian tube epithelium, inclusion cysts and serous cystadenomas with a Müllerian phenotype expressed high levels of *LGR5,* and *LGR5* expression was restricted to PAX8^+^/FOXJ1^−^ secretory cells of the tubal epithelium. Strong stromal *LGR5* expression without epithelial *LGR5* expression was consistently observed in the path from serous cystadenoma to serous borderline tumor to low grade serous carcinoma (LGSC). Unlike LGSC, high grade serous carcinoma (HGSC), clear cell carcinoma, endometrioid carcinomas displayed various epithelial-stromal *LGR5* expression. Notably, high levels of *LGR5* expression were observed in serous tubal intraepithelial carcinoma, which slightly declined in invasive HGSC. *LGR5* expression was significantly associated with improved progression-free survival in HGSC patients. Moreover, in vitro assays demonstrated that LGR5 expression suppressed tumor proliferation and migratory capabilities. Taken together, these findings indicate a tumor-suppressive role for *LGR5* in the progression of HGSC.

## Introduction

Ovarian epithelial tumors are heterogeneous neoplasms primarily classified according to cell type and are subdivided into benign, borderline, and malignant based on the degree of cellular proliferation and atypia, and the presence of stromal invasion^1^. Ovarian cancer has the lowest five year survival rate among gynecologic cancers at 46%^2^. Over 70% of patients are diagnosed with advanced-stage disease^3^ and their five-year survival rates are only 29%, by contrast with 92% for early-stage disease^4^. Annually worldwide, 314 000 women are diagnosed and 207 000 die of ovary cancers^5^. Carcinomas are the most common ovarian cancers accounting for 90% of cases, and five main types have been identified: high grade serous carcinoma (HGSC), endometroid carcinoma (EC), clear-cell carcinoma (CCC), mucinous carcinoma (MC), and low-grade serous carcinoma (LGSC)^6^. Depending on their clinicopathological features and molecular profile, these subtypes can be classified either type 1 or type 2 tumors. Type 1 carcinomas include clear cell, endometrioid, mucinous, and LGSC, while type 2 carcinomas mainly comprise HGSCs^7^. Type 2 carcinomas are more frequently detected in advanced stage, and display a universal *TP53* mutations^7^.

*Lgr5* has been identified as an adult stem cell marker in multiple organs such as small and large intestines, stomach, hair follicle, kidney, breast, and liver^8–13^. *Lgr5*^+^ stem cells have been demonstrated to be the cells of origin of multiple tumor types in the stomach, intestine, and liver^14–16^. Furthermore, *Lgr5*^+^ cancer cells have been shown to serve as so-called cancer stem cells in growing cancer tissues^17,18^. Lineage tracing studies in the mice have revealed that multiple local *Lgr5*^+^ cells contribute to homeostatic maintenance and rapid restoration of the surface epithelia during ovulatory damage, suggesting *Lgr5* as a marker of stem/progenitor populations of the ovary and tubal epithelia^19^. In addition, *Lgr5*-expressing ovary surface epithelium (OSE) stem cells exhibit increased tumorigenicity in a TP53- and RB1-deficient background^20^. These findings clearly suggest that the resident *Lgr5*^+^ stem cells reside in the OSE and may constitute the cell of origin of epithelial ovarian tumors^19,20^.

In contrast to the remarkable discoveries from mouse studies, there has been little investigation exploring *LGR5* expression in human ovary/oviduct or ovarian epithelial tumors. To our knowledge, only two studies have examined *LGR5* expression in human ovarian tissues. Ng et al. demonstrated the presence of *LGR5* transcripts in OSE and tubal epithelia using fluorescence in situ hybridization (ISH) in the human ovary and distal fallopian tube^19^, but *LGR5* expression in ovarian tumors was not explored. Amsterdam et al. examined LGR5 expression using IHC in normal ovaries, borderline tumors, and serous carcinomas and reported that LGR5 expression was present in normal OSE, but not in ovarian tumors^21^. However, it is well known that RNA ISH is a much more reliable method for detecting *LGR5* expression in human FFPE specimens than IHC. Therefore, in this study we employed the RNA ISH and aimed to thoroughly investigate the expression profile of *LGR5* using RNA ISH in normal human ovary and fallopian tubes as well as various benign and malignant ovarian epithelial tumors.

## Material and methods

### Tissue samples

This study included the following formalin-fixed and paraffin-embedded (FFPE) ovarian borderline tumor and carcinoma samples collected from the patients who underwent surgical resection at Seoul National University Hospital (SNUH) (Seoul, Korea) from 2010 to 2021; LGSC (n = 12), HGSC (n = 64), endometrioid carcinoma (EC, n = 47), clear cell carcinoma (CCC, n = 48), mucinous carcinoma (MC, n = 44), STIC lesions (n = 21), serous borderline tumor (SBT, n = 7), and mucinous borderline tumor (MBT, n = 7). STIC lesions were identified entirely from sampled fallopian tubes according to the ‘sectioning and extensively examining the fimbriated end’ (SEE-FIM) protocol^22^. The classification of histological subtypes of carcinomas was independently determined by two pathologists (C.L. and J.K.M.). For HGSC cases, clinicopathological data including age, FIGO stage, family history, serum CA-125 levels, BRCA1 and BRACA 2 mutation status, time of death, tumor recurrence, and follow-up time were obtained by reviewing the clinical and pathologic reports^23^. For the validation study, a total of 1104 ovarian serous carcinoma patients were included from the Kaplan–Meier-plotter datasets (http://kmplot.com/analysis). We also collected FFPE normal ovary/fallopian tube and benign lesion samples from the patients who underwent surgical resection at Jeju National University Hospital (JNUH) (Jeju, Korea) from 2018 to 2021. This study was approved by the Institutional Review Board (IRB) of JNUH (2021–07-007) and SNUH (H-2202–031-1297). The IRB confirmed that informed consent for FFPE samples was waived due to the retrospective nature of the study. All procedures were performed in accordance with the ethical standards of the Helsinki Declaration of 1964 and subsequent versions.

### Tissue microarray (TMA) construction

Three TMAs containing 64 HGSC cores and 47 MC cores were previously assembled^23^. In total, 9 TMAs were newly constructed including normal ovaries and fallopian tubes as well as a variety of benign and malignant lesions. In brief, a single representative area comprising more than 70% of tumor cell population was identified through microscopic examination and marked on a hematoxylin and eosin (H&E) slide. Core tissue cylinders with a 4 mm in diameter were collected from an individual FFPE specimen of paraffin block and arranged in a new recipient paraffin block using a trephine apparatus (SuperBioChips Laboratories, Seoul, Korea).

### Immunohistochemistry and interpretation

Immunohistochemistry (IHC) for P53, PAX8, FOXJ1, estrogen receptor and β-catenin was performed on 4-μm TMA sections using a BOND-MAX automated immunostainer and a Bond Polymer Refine Detection kit (Leica Microsystems, Wetzlar, Germany) according to the manufacturer’s guidelines. The primary antibodies were anti-P53 (DAKO, 1:1000), anti-PAX8 (Proteintech, 1:300), anti-FOXJ1 (Invitrogen, 1:100), anti-estrogen receptor (DAKO, 1:100), and anti-β-catenin (BD Transduction, 1:800). Estrogen receptor and β-catenin were considered positive when more than 10% of tumor cell nuclei were strongly stained.

### RNA in situ hybridization and interpretation

RNA in situ hybridization (ISH) performed using RNAscope FFPE assay kit (Advanced Cell Diagnostics, Hayward, CA) as previously described^24^. Briefly, 4-μm FFPE tissue sections are pretreated with heat and protease digestion followed by hybridization with the probe. Then, an HRP-based signal amplification system was hybridized to a probe before color development with 3,3′-diaminobenzeidine tetrahydrochloride (DAB). Cases with UBC easily visible under a 10 × objective lens were considered to be adequate according to the manufacturer’s recommendation. Positive staining was indicated by brown punctate dots in the nucleus and/or cytoplasm. *LGR5* transcripts were quantified according to the manufacturer’s scoring guidelines: score 0, no staining; score 1: one to three dots per cell; score 2: four to 10 dots per cell; score 3: more than 10 dots per cell; score 4: more than 15 dots per cell. The housekeeping gene ubiquitin C (UBC) and the bacterial gene DapB served as positive and negative controls, respectively. The histo-scores (H-scores) were calculated as follows: RNAscope score × % of positive cells, ranging from 0 to 400. For statistical analyses, H-score of 40 was chosen based on the median (H-score: 20) and mean (H-score: 56) values of *LGR5* H-scores; the tumor was considered high (H-score > 40) when more than 20% of tumor cells express *LGR5* with a score 2 or higher.

### Combined RNA ISH and multiplex IHC

We performed a combined RNA ISH and multiplex IHC by sequentially performing RNA ISH and multiple IHC on identical TMA slides as previously described^25^. Briefly, RNA ISH for *LGR5* was first performed, followed by scanning and image acquisition of the entire TMA slide using an Aperio AT2 scanner (Leica Biosystems, Newcastle upon Tyne, UK). Then, the slide was subjected to IHC for FOXJ1 staining. After scanning the newly stained slide, it was treated with stripping buffer (20% sodium dodecyl sulfate, 0.5 M Tris–HCl pH 6.8, β-mercaptoethanol, and distilled water) and microwaved to perform additional stripping. After antigen retrieval, IHC for PAX8 staining was performed on the same slide followed by scanning. For analysis, each 4-mm TMA core of the virtual TMA slides was extracted using an Aperio ImageScope (Leica Biosystems). CellProfiler (ver. 3.1.8. Broad Institute, Cambridge, MA) was used to further process the core images. All core images from each staining procedure were converted into grayscale images and each positive expression within the cores was further converted to a specific color; blue for *LGR5*, red for PAX8 IHC, and green for FOXJ1 IHC. For each core, three pseudocolor images representing the three stains were aligned and merged into a single image. DAB staining of RNA ISH for *LGR5* is irremovable and therefore remains during subsequent IHC and scanning procedures. As a result, *LGR5* expression remains in all three scanned images and appears white when the three-color images are merged.

### Cell lines and transfection

Four human HGSC cell lines (CaoV-3, NIH OVCAR-3, SNU-8, and SNU-119) were purchased from the Korean Cell Line Bank (Seoul, Korea). Cells were cultured in RPMI 1640 medium (Welgene, Daegu, Korea) containing 10% fetal bovine serum (FBS) (Gibco, Carlsbad, CA) and 1% penicillin/streptomycin (Gibco) and maintained at 37 °C in a humidified incubator with 5% CO2. Full-length cDNA encoding LGR5 (pEX-LGR5) and control vector were purchased from GeneCopoeia (Rockville, MD). Cancer cells were seeded at 1 × 10^5^ cells/well in 6-well plates after transfection with control vector or pEX-LGR5 (2.5 μg) using the Neon transfection system (Thermo Fisher Scientific). One or two days after transfection, the cells were subjected to real-time PCR, immunoblotting or functional assays.

### Western blot assay

Cellular proteins were extracted in lysis buffer (iNtRON Biotechnology, Seongnam, Korea) and quantified using BCA protein assay kits (Pierce, Rockford, IL, USA). Cell lysates were run on a 10% SDS–polyacrylamide gel and were transferred to a PVDF membrane (Millipore Corporation, Bedford, MA, USA). The membrane was blocked in 5% nonfat dry milk in PBS-Tween-20 (0.1%, *v/v*) for 1 h and incubated with primary antibodies overnight at 4 °C. After washing with TBS containing 0.1% Tween-20, the membrane was incubated for 1 h with secondary antibodies. Alliance-Mini. An HD9 chemiluminescence documentation system (UVItec Cambridge, UK) was used to visualize target proteins. Anti-LGR5 (ab238518) and anti–GAPDH (catalog number: #TA505454) antibodies were purchased from Abcam and Origene, respectively. Anti-ERK (catalog number: #4695), anti-AKT (catalog number: #4691), anti-phospho-ERK (catalog number: #4370), anti-phospho-AKT (catalog number: #4060), anti-cleaved PARP (catalog number: #5625), anti-cleaved caspase-3 (catalog number: #9661) and anti-BIM (catalog number: #2933) antibodies were purchased from Cell Signaling Technology (Danvers, MA, USA).

### Proliferation assay

Cells were harvested 1 day after transfection, seeded at 5 × 10^3^ cells/well in a 96-well plate and incubated at 37 °C. At the indicated time points, cell growth was evaluated by adding 10 μl of Cell Counting Kit-8 reagent (Dojindo, Kumamoto, Japan) into each well and incubating for an hour. Absorbance was measured at 450 nm using a spectrophotometer (Thermo Labsystems, Rockford, IL, USA).

### Caspase‑3 activity assay

Caspase-3 enzymatic activity was measured using a Caspase-Glo 3 Assay Kit (Promega, Madison, WI, USA, catalog number: G8091). After transfection with control or LGR5-containing vector, OVCAR-3 or SNU-8 cells (1 × 10^4^ cells/mL) were seeded and cultured in 96-well plates (100 μL/well) for 24 h in triplicate. Caspase-Glo Reagent (100 μL/well) was added to each well and the cells were incubated in the dark at room temperature for 3 h on a shaker. The luminescence in each well was measured using the GloMax Navigator System (Promega).

### Migration assay

Cells were starved in serum-free RPMI medium for three hours. After starvation, the cells were harvested with trypsin treatment and resuspended. Twenty four–well culture plates were divided into upper and lower wells by a transwell insert (pore size, 8 mm) (BD Bioscience, San Diego, CA). The upper surface of the transwell was loaded with 2 × 10^5^ cells in 300 μL serum-free RPMI medium and the lower wells contained 500 μL RPMI with 10% FBS. After 24 h of incubation, nonmigrated cells were removed from the top of each insert using a cotton swab. Migrated cells on the bottom surface were fixed in methanol for 10 min and counted after staining with crystal violet for one hour. All experiments were independently performed at least two to three times.

### Statistical analysis

Statistical analyses were performed using SPPSS software version 18.0 (SPSS, Chicago, IL) and Prism version 9.0.0 (GraphPad Software, San Diego, CA; https://www.graphpad.com/scientific-software/prism). Between-group comparisons of *LGR5* H-scores in epithelial or stroma cells were performed using Student’s t test or Turkey’s multiple comparisons test. Correlations between *LGR5* expression and clinicopathological parameters were assessed using Pearson’s chi-square test. Survival curves were estimated using the Kaplan–Meier method, and the log-rank test was used to compare groups. A *P* value < 0.05 was considered statistically significant.

### Ethics approval

This study was approved by the Institutional Review Board of JNUH (2021-07-007) and SNUH (H-2202-031-1297). The institutional Review Board confirmed that informed consent for FFPE samples was waived because of the retrospective nature of the study. All procedures were performed in accordance with the ethical standards of the Helsinki Declaration of 1964 and its subsequent versions.

## Results

### *LGR5* expression in normal ovary and inclusion cysts

**Figure 1:**
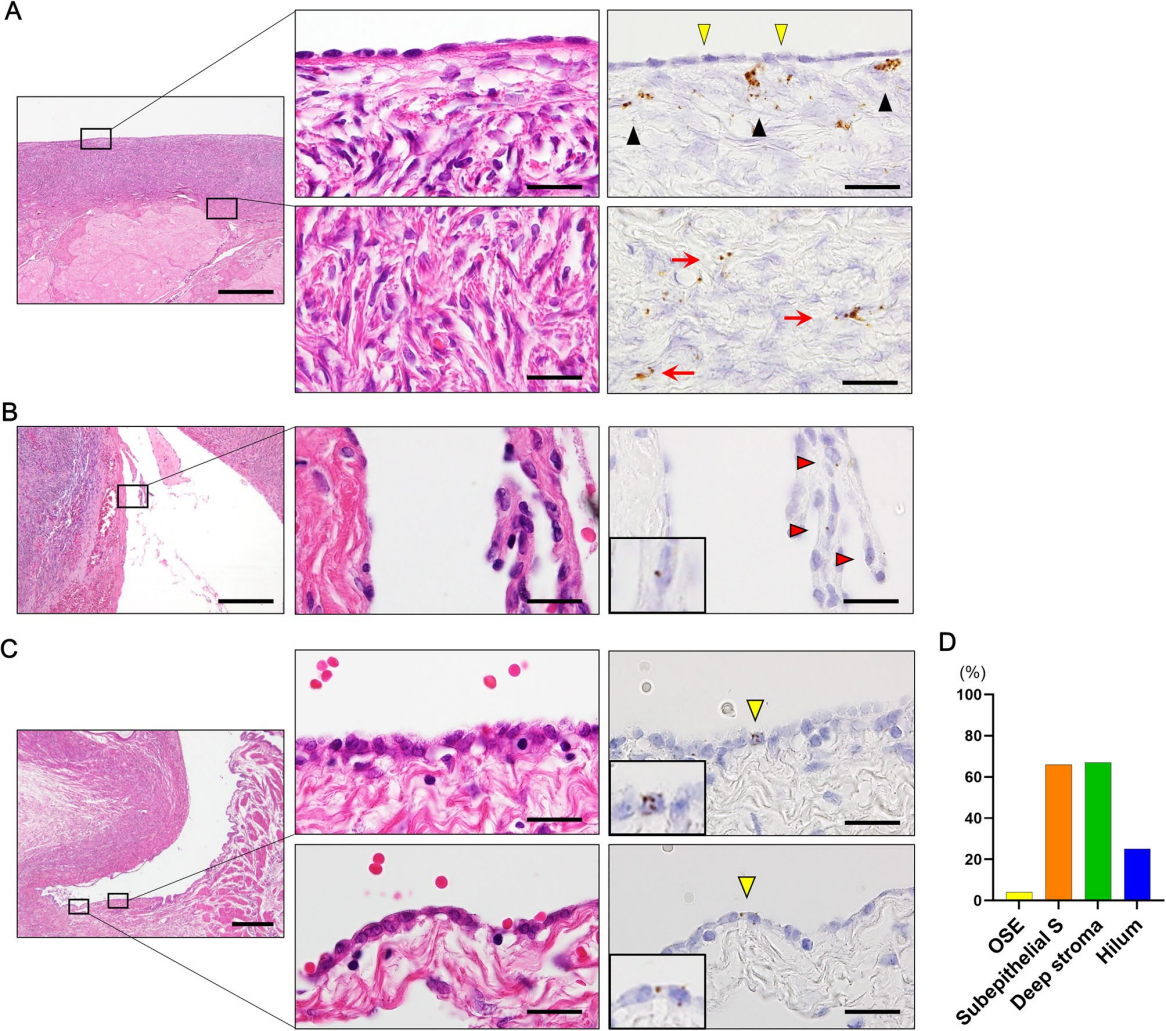
*LGR5* expression in normal ovary surface epithelium and stromal cells. (**A**) *LGR5* expression is not observed in the ovary surface epithelium (OSE) (indicated by yellow arrowheads), while subepithelial stromal cells often exhibit *LGR5* expression (indicated by black arrowheads). Sparse *LGR5*-positive stromal cells are observed in the deep ovarian stroma (indicated by red arrows). Scale bar, 0.5 mm (left panel) and 20 µm (middle and right panels). (**B**) Only one ovary displayed *LGR5*-positive surface epithelial cells, which appeared among proliferating epithelial cells near the hemorrhagic area (indicated by red arrowheads, magnified image shown in the inset). Scale bar, 0.5 mm (left panel) and 20 µm (middle and right panels). (**C**) *LGR5*-positive cells are rarely observed in the ovarian hilum (indicated by yellow arrowheads, magnified image shown in the insets). Scale bar, 0.5 mm (left panel) and 20 µm (middle and right panels). (**D**) A bar graph showing the percentage of cases in which *LGR5* expression was observed in each area of the ovary (OSE, n = 23, subepithelial S, n = 23, deep stroma, n = 29, hilum, n = 4). *Subepithelial S* Subepithelial stroma.

Before applying RNAscope to FFPE human ovary pathologies, we tested its ability to detect *LGR5* transcripts using ovarian mature teratoma samples and confirmed its specificity by demonstrating *LGR5*-positive cells in the bulb and outer root sheath of hair follicles, which is a well-known location for *LGR5* cells (Supplementary Fig. 1). Then, we performed RNA ISH to examine *LGR5* expression in a number of normal human ovaries (n = 15, 29 spots): ovaries with (8 spots) and without (21 spots) epithelial proliferation. Normally, ovary surface epithelium (OSE) exhibits no *LGR5* expression (Fig. 1A). Notably, however, *LGR5* transcripts were frequently observed in ovarian stromal cells: subepithelial (65%, 15 of 23 spots) and deep areas (66%, 19 of 29 spots). Expression intensity was very weak in most cases, but slightly higher in subepithelial areas than in deep areas (Fig. 1A). Only one case displayed a few *LGR5*-positve cells in the OSE, which were found within papillary proliferative cells. Hemorrhage and fibrosis were observed nearby, suggesting a regenerative process (Fig. 1B). In mouse studies, the hilum was also demonstrated to be the primary location wherein *LGR5*-positive stem cells reside. Therefore, we explored hilar areas (n = 4) and observed *LGR5*-expressing cells in one case, in which the number of positive cells and their expression levels were extremely low (Fig. 1C). The overall frequency of *LGR5* expression regardless of intensity is presented in Fig. 1D, clearly showing that *LGR5* is normally more often expressed in ovarian stromal cells than in epithelial cells.

**Figure 2:**
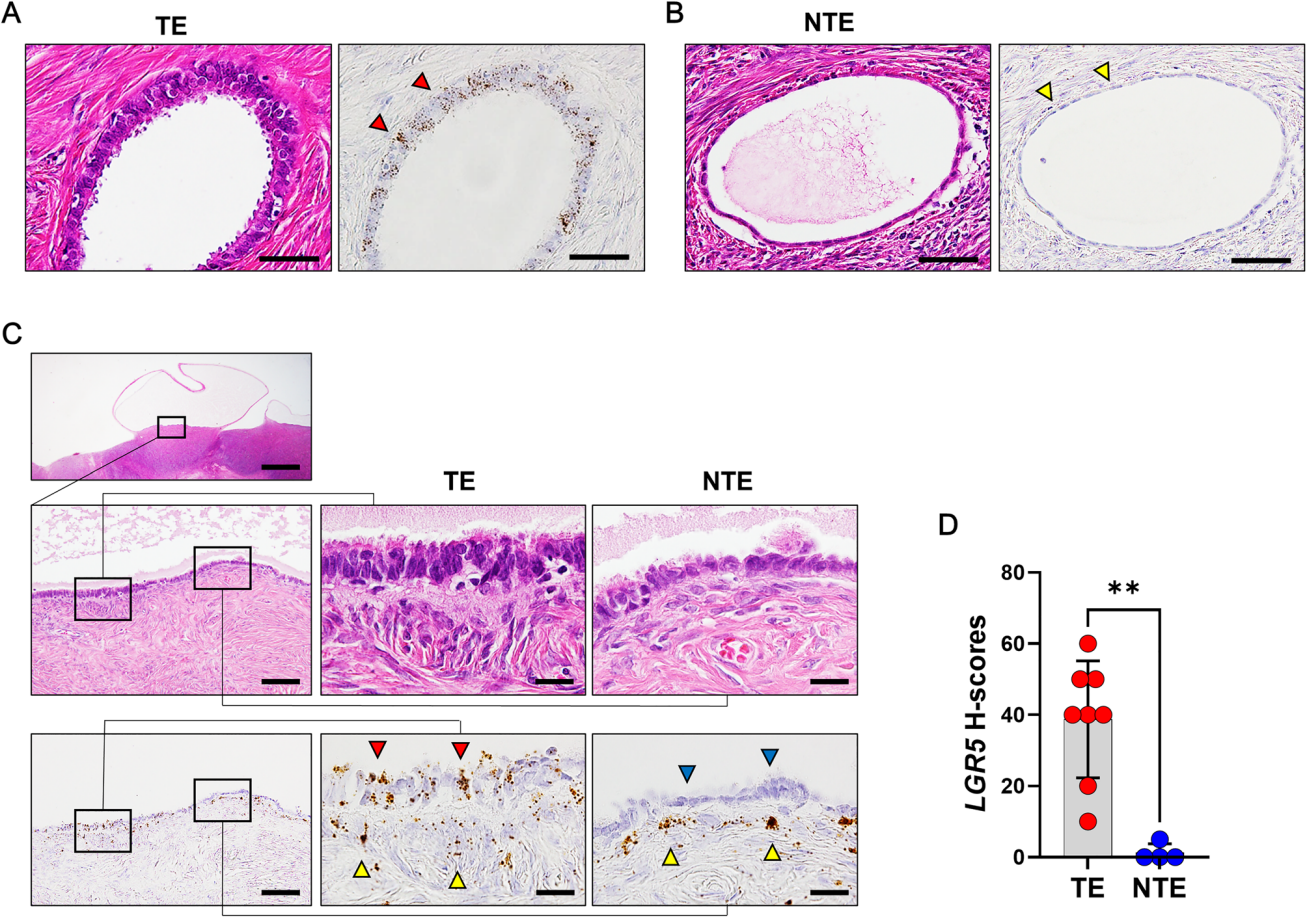
*LGR5* expression in the inclusion cysts**.** (**A**) The tubal type epithelium (TE) of an inclusion cyst showing a significant level of *LGR5* expression (indicated by red arrowheads). Scale bar, 50 µm. (**B**) Inclusion cysts that have nontubal type epithelium (NTE) express no or negligible levels of *LGR5* (indicated by yellow arrowheads). Scale bar, 50 µm. (**C**) An inclusion cyst that contains both TE and NTE. *LGR5* expression is observed in the TE (indicated by red arrow heads) along with strong *LGR5* expression in subepithelial stromal cells (indicated by yellow arrowheads), whereas NTE does not express *LGR5* (indicated by blue arrowheads). Scale bar, 0.5 mm (left upper panel), 0.2 mm (left middle and lower panels) and 20 µm (middle and right panels). (**D**) A bar graph showing histo-scores (H-scores) of *LGR5* in the inclusion cysts with TE (n = 8) or NTE (n = 4). **P < 0.01 by unpaired t test.

Next, we evaluated *LGR5* expression in Inclusion cysts (ICs) (n = 12) because they are common benign lesions in the ovary and are considered a precursor for serous carcinomas. ICs are mostly lined by tubal-type epithelium (TE) or simple cuboidal or flat cells resembling ovarian surface epithelium (hereafter referred to as nontubal-type epithelium, NTE). Remarkably, ICs with TE exhibited high levels of *LGR5* expression (Fig. 2A), whereas those with NTE expressed no or little *LGR5* (Fig. 2B). Interestingly, there was a protruding cystic lesion, in which TE and NTE were observed in a mixed pattern and only TE expressed *LGR5*, while subepithelial *LGR5* expression was observed beneath both types of epithelium (Fig. 2C), suggesting that epithelial *LGR5* expression is associated with a Müllerian phenotype. Indeed, there was a significant difference in histo-scores (H-scores) for *LGR5* between ICs with TE (n = 8) and those with NTE (n = 4), which were much higher in the ICs with TE (mean ± SD: 38.75 ± 16.42) than in those with NTE (mean ± SD: 1.25 ± 2.50) (*P* < 0.01, Fig. 2D).

### *LGR5* expression in the low-grade serous carcinogenesis

**Figure 3:**
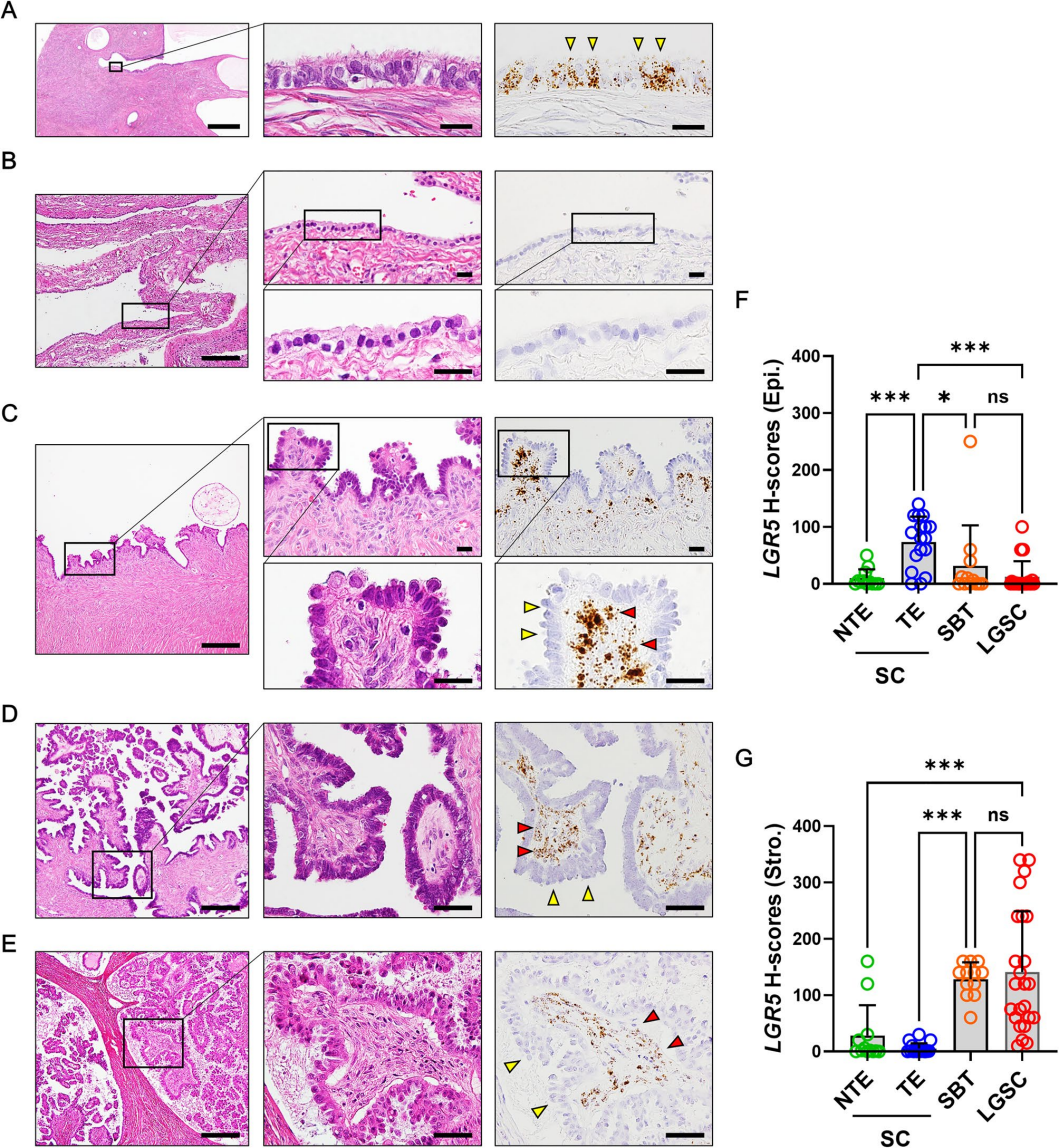
*LGR5* expression in serous cystadenoma (SC), serous borderline tumor (SBT), and low-grade serous carcinoma (LGSC). SCs are lined by a single layer of columnar, ciliated cells resembling normal tubal type epithelium (TE) or flat, cuboidal nontubal type epithelium (NTE). (**A**) High levels of *LGR5* expression are detected in most cases of TE in SCs. Scale bar, 0.5 mm (left panel) and 20 µm (middle and right panels). (**B**) NTE in SCs mostly exhibits little or no *LGR5* expression. Scale bar, 0.2 mm (left panel) and 20 µm (middle and right panels). (**C**) In an SC with focal papillary tufting, subepithelial stromal cells express high levels of *LGR5*, whereas nontubal epithelial cells exhibit no *LGR5* expression. Scale bar, 0.2 mm (left panel) and 20 µm (middle and right panels). Strong stromal *LGR5* expression was observed in most SBTs (**D**) and LGSCs (**E**). Scale bar, 0.2 mm (left panel) and 50 µm (middle and right panels). Bar graphs showing the histo-scores (H-scores) of *LGR5* in epithelial tumor cells (**F**) and stromal cells (**G**) in SC (n = 16, spots = 29), SBT (n = 7, spots = 14), and LGSC (n = 9, spots = 18). Yellow arrowheads indicate epithelial tumor cells, and red arrowheads indicate stromal cells. The data are shown as means ± SD. *ns* not significant. **P < 0.01, ****P < 0.0001 by Tukey’s multiple comparisons test.

Ovarian serous carcinomas develop through two distinct molecular pathways; type 1 and type 2. LGSC is believed to arise from a serous cystadenoma (SC) or serous adenofibroma which progresses to serous borderline tumor (SBT), and then to invasive micropapillary serous carcinoma in a slow stepwise fashion (type 1)^26^. Therefore, we investigated the expression profile of *LGR5* over the type 1 carcinogenesis pathway in 16 SCs, 7 SBTs, and 9 LGSCs. Similar to inclusion cysts, SCs are also lined by a single layer of columnar and ciliated TE or flat/cuboidal NTE. *LGR5* expression was observed in the most SCs with TE (15 of 17 spots, H-scores [mean ± SD]: 75.0 ± 45.8) (Fig. 3A). In contrast, in SCs lined by NTE, *LGR5* expression was less often observed in the tumor epithelial cells with much lower H-scores (7 of 12 spots, [mean ± SD]: 3.9 ± 6.8) (Fig. 3B). One of the major histological differences between TE and NTE in SCs is the presence of ciliated cells. By performing serial staining for paired box gene 8 (PAX8, a secretory cell marker) and forkhead box J1 (FOXJ1, a ciliated cell marker), we demonstrated that TE and NTE both express PAX8, while only TE displays FOXJ1 expression. (Supplementary Fig. 2). We found an interesting case of SC with NTE, in which focal papillary proliferation occurs and strong *LGR5* expression was observed in the subepithelial stromal cells with no or little epithelial *LGR5* expression similar to the pattern we observed in some normal ovary samples or ICs (Fig. 3C). Remarkably, this distinct expression pattern was observed in the majority of SBTs (6 of 7 cases, Fig. 3D and Supplementary Fig. 3), and it remained a predominant pattern in LGSC (8 of 9 cases, Fig. 3E). The H-scores of epithelial and stromal *LGR5* are shown as scatter plots in Fig. 3F,G, demonstrating the increasing stroma-localized *LGR5* expression during low-grade serous carcinogenesis.

### *LGR5* expression in the normal fallopian tubes

**Figure 4:**
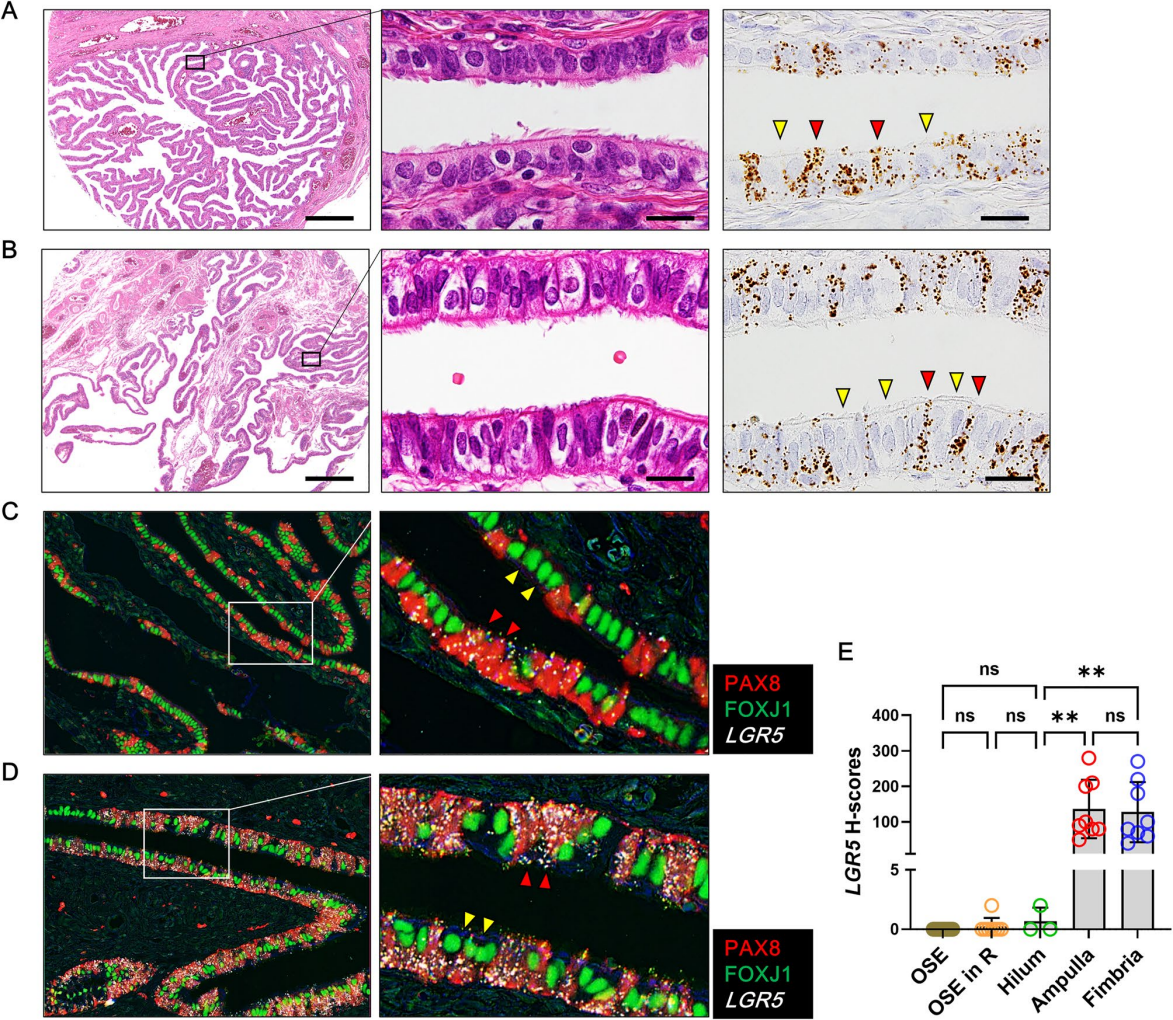
High levels of *LGR5* expression in the fallopian tubes. (**A**) Ampulla of the fallopian tube harbors two types of epithelial cells: ciliated cells (indicated by yellow arrowheads) and secretory cells (indicated by red arrowheads). Strong *LGR5* expression seems to be confined to secretory cells. Scale bar, 0.5 mm (left panel) and 50 µm (middle and right panels). (**B**) The epithelium in the fimbria exhibits the same *LGR5* expression pattern as in the ampulla. Scale bar, 0.5 mm (left panel) and 50 µm (middle and right panels). Combined in situ hybridization for *LGR5* (white dots) and multiplex immunohistochemistry for PAX8 (red nuclear stain) and FOXJ1 (green nuclear stain) was performed on a ciliated cell-rich area (**C**) and on a secretory cell-rich area (**D**). PAX8-positive secretory cells display strong *LGR5* expression (indicated by red arrowheads), while FOXJ1-positive ciliated cells do not express *LGR5* (indicated by yellow arrowheads)*.* (**E**) A bar graph showing that histo-scores (H-scores) of *LGR5* are much higher in fallopian tubes than in ovaries. OSE in R, ovary surface epithelium in regeneration. The data are shown as the means ± SD. *ns* not significant. **P < 0.01 by Tukey’s multiple comparisons test.

With the discovery of high levels of *LGR5* expression in TE in both ICs and SCs, we examined *LGR5* expression in 8 pairs of ampulla and fimbriae of fallopian tubes and found that *LGR5* was highly expressed in all cases examined. A large number of *LGR5*-positive cells with strong intensity were diffusely distributed both in the ampulla (Fig. 4A) and fimbriae (Fig. 4B). In addition, high-power view examination indicated that *LGR5* expression seems to be restricted to nonciliated cells. To specifically identify cells expressing *LGR5*, we combined RNA ISH (for *LGR5*) and multiplex immunohistochemistry (for PAX8 and FOXJ1); PAX8 stains nonciliated secretory cells and FOXJ1 stains ciliated cells in the tubal epithelium as mentioned earlier. Remarkably, *LGR5* was expressed only in PAX8-positive cells, but not in FOXJ1-positive cells (Fig. 4C,D). We measured *LGR5* H-scores in 8 fallopian tube samples, which were compared to those in normal ovaries, revealing much higher levels of *LGR5* expression in the fallopian tube epithelium than in ovaries (Fig. 4E).

### *LGR5* expression in the high-grade serous carcinogenesis

**Figure 5:**
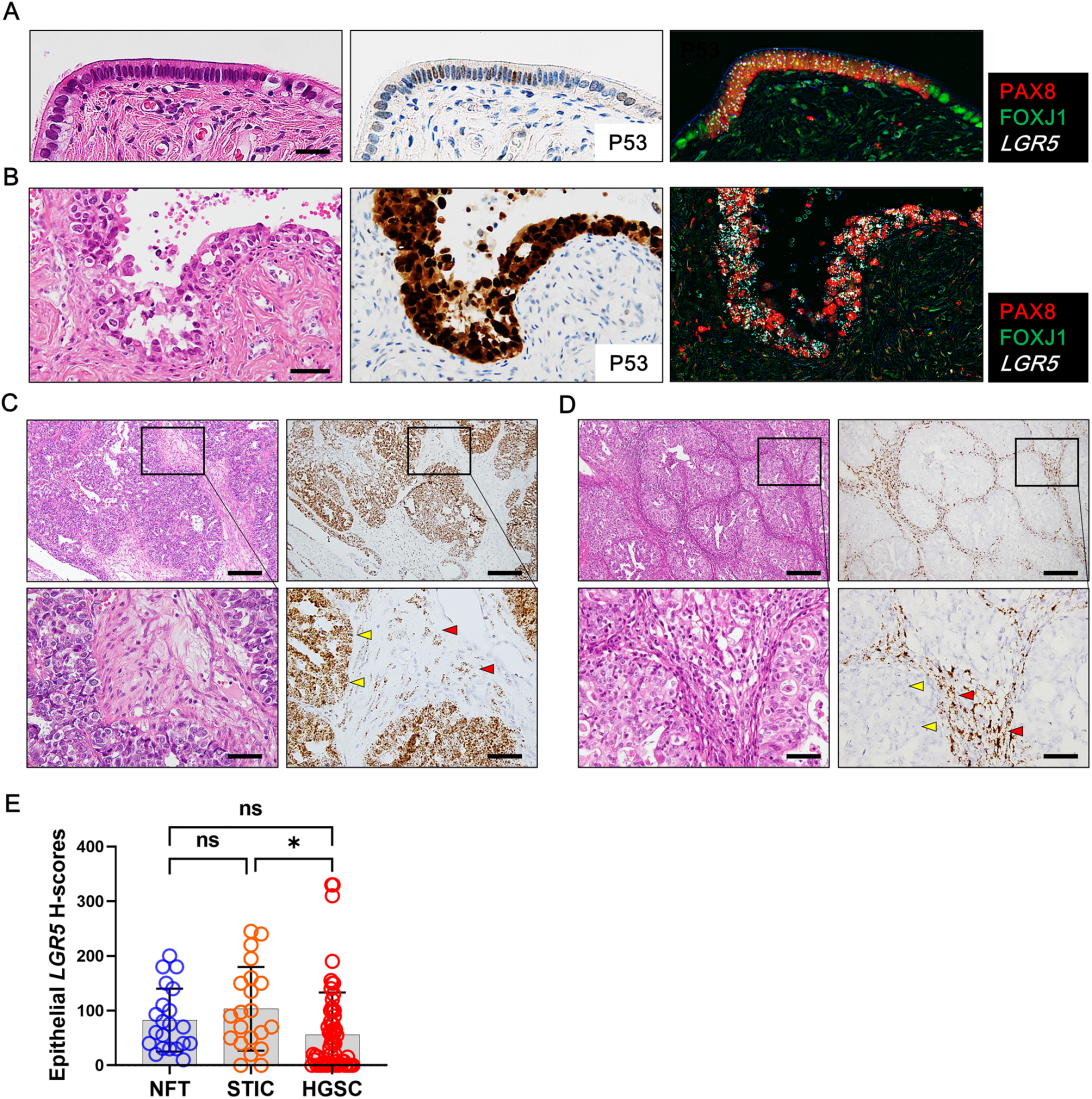
*LGR5* expression in secretory cell outgrowth (SCOUT), serous tubal intraepithelial carcinoma (STIC) and high-grade serous carcinoma (HGSC). Combined in situ hybridization for *LGR5* and immunohistochemistry for PAX8 and FOXJ1 shows increased *LGR5* expression in the SCOUT (**A**) and STIC (**B**) lesions in which PAX8-positive cells proliferate. (**C**) Representative images of *LGR5* expression in HGSC. *LGR5* expression is frequently observed not only in carcinoma cells but also in adjacent stromal cells. Scale bar, 0.5 mm (upper panels) and 50 µm (lower panels). (**D**) In some cases, *LGR5* expression is only detected in stromal cells. Scale bar, 0.5 mm (upper panels) and 50 µm (lower panels). (**E**) A bar graph showing the histo-scores (H-scores) of *LGR5* in in the nontumor fallopian tubes (NFTs) adjacent to STIC lesions (n = 21), STIC lesions (n = 21), and HGSC lesions (n = 64). The data are shown as the means ± SD. ns, not significant. *P < 0.05 by Tukey’s multiple comparisons test.

It is believed that most HGSCs originate from fallopian tube secretory cells with a stepwise progression of tubal epithelium to precursor lesions to carcinoma, known as the type 2 pathway^27^. Precursor lesions include secretory cell outgrowth (SCOUT), p53 signature, and serous tubal intraepithelial carcinoma (STIC)^27^. To investigate the *LGR5* expression profile of *LGR5* throughout type 2 carcinogenesis, we collected one SCOUT and 21 STIC lesions. Unfortunately, we were not able to obtain P53 signature lesion. Combined analysis demonstrated that PAX8-positive secretory cells expanded and expressed high levels of *LGR5* expression in SCOUT (Fig. 5A) and STIC lesions (Fig. 5B). Among 21 STICs, 19 cases expressed *LGR5*, and 9 cases exhibited higher *LGR5* H-scores than adjacent nontumorous tubal epithelium (NTTE) (Supplementary Fig. 4). Next, we performed RNA ISH for *LGR5* on tissue microarrays containing 64 HGSCs. In total, 30 cases (47%) of carcinoma cells expressed significant levels of *LGR5*. It was common to find the cases where *LGR5* expression was observed not only in carcinoma cells, but also in stromal cells (10 cases, 16%) (Fig. 5C). In addition, there were cases where *LGR5* was expressed exclusively in stromal cells (11 cases, 17%) as seen in SBT or LGSC (Fig. 5D). When comparing *LGR5* H-scores, there was no significant difference in *LGR5* expression between NTTE and STIC lesions, while *LGR5* H-scores were slightly lower in HGSCs than in STICs (Fig. 5E). Since HGSCs are characterized by P53 mutation, we examined whether the P53 mutation type (overexpression vs. null type) is associated with *LGR5* expression. However, no difference was observed in *LGR5* H-scores between these subgroups (Supplementary Fig. 5).

To investigate whether there is any association between *LGR5* and clinico-pathological-molecular characteristics in HGSCs, we classified HGSCs into *LGR5*-low and *LGR5*-high groups with an H-score cutoff of 40. This analysis was separately performed for epithelial and stromal *LGR5* expression, and the results are summarized in Table 1. Thirty cases (47%) of HGSCs belonged to epithelial *LGR5*-high group, which was more frequently observed in the elderly (≥ 55 yr) (*P* = 0.001). High *LGR5* expression in carcinoma cells was associated with low CA-125 levels (*P* = 0.041) and was negatively correlated with recurrence rate (*P* = 0.013). On the other hand, epithelial *LGR5* expression exhibited no significant associations with other characteristics such as family history of ovarian cancer, menopause, FIGO stage, platinum sensitivity, germline BRCA1 and 2 mutations, or estrogen receptor positivity. High stromal *LGR5* expression was observed in 21 (33%) cases and showed associations with younger age (*P* = 0.026), family history of breast cancer (*P* = 0.011), and the presence of residual tumor (*P* = 0.006).

**Table 1:** Association between *LGR5* and clinicopathological characteristics in high-grade serous carcinomas.

Characteristics	No. of cases (%)	Epithelial *LGR5*		*P-*value^#^	Stromal *LGR5*		*P-*value^#^
		Low (%)	High (%)		Low (%)	High (%)	
	64 (100)	34 (53)	30 (47)		43 (67)	21 (33)	
**Age (yr.)**							
< 55	33 (52)	24 (73)	9 (27)	0.001	18 (55)	15 (45)	0.026
≥ 55	31 (48)	10 (32)	21 (67)		25 (81)	6 (19)	
**Family history of breast cancer**							
Absent	61 (95)	32 (52)	29 (48)	0.63	43 (71)	18 (29)	0.011
Present	3 (5)	2 (67)	1 (33)		0 (0)	3 (100)	
**Family history of ovarian cancer**							
Absent	61 (95)	32 (52)	29 (48)	0.63	41 (67)	20 (33)	0.984
Present	3 (5)	2 (67)	1 (33)		2 (67)	1 (33)	
**Menopause**							
No	25 (39)	16 (64)	9 (36)	0.163	14 (56)	11 (44)	0.127
Yes	39 (61)	18 (46)	29 (54)		29 (74)	10 (26)	
**Serum CA-125, IU/ml**							
< 700	34 (53)	14 (41)	20 (59)	0.041	24 (71)	10 (29)	0.537
≥ 700	30 (47)	20 (67)	10 (33)		19 (63)	11 (37)	
**FIGO stage**							
1–2	5 (8)	3 (60)	2 (40)	0.918	2 (40)	3 (60)	0.269
3	41 (64)	22 (54)	19 (46)		27 (66)	14 (34)	
4	18 (28)	9 (50)	9 (50)		14 (78)	4 (22)	
**Malignant ascites**							
No	7 (11)	4 (57)	3 (43)	0.308	4 (57)	3 (3)	0.712
Yes	55 (86)	30 (55)	25 (45)		38 (69)	17 (31)	
Unknown	2 (3)	0 (0)	2 (100)		1 (50)	1 (50)	
**Residual tumor after operation**							
Absent	43 (67)	23 (54)	20 (47)	0.934	24 (56)	19 (44)	0.006
Present	21 (33)	11 (52)	10 (48)		19 (91)	2 (9)	
**Recurrence**							
No	30 (47)	11 (37)	19 (63)	0.013	18 (60)	12 (40)	0.250
Yes	34 (53)	23 (68)	11 (32)		25 (74)	9 (26)	
**Platinum sensitivity**							
Sensitive	53 (83)	30 (57)	23 (43)	0.247	38 (72)	15 (28)	0.331
Resistant	9 (14)	4 (44)	5 (56)		5 (56)	4 (44)	
Not applicable	2 (3)	0 (0)	2 (100)		0 (0)	2 (100)	
**Germline *BRCA1* mutation**							
Absent	44 (69)	24 (55)	20 (45)	0.736	29 (66)	15 (34)	0.747
Present	20 (31)	10 (50)	10 (50)		14 (70)	6 (30)	
**Germline *BRCA2* mutation**							
Absent	57 (89)	31 (54)	26 (46)	0.564	38 (67)	19 (33)	0.800
Present	7 (11)	3 (43)	4 (57)		5 (71)	2 (29)	
**Germline *BRCA1* or *2* mutations**							
Absent	38 (59)	21 (55)	17 (45)	0.679	25 (66)	13 (34)	0.773
Present	26 (41)	13 (50)	13 (50)		18 (69)	8 (31)	
**Estrogen receptor**							
Negative	24 (37)	12 (50)	12 (50)	0.698	26 (65)	14 (35)	0.630
Positive	40 (63)	22 (55)	18 (45)		17 (71)	7 (29)	

*No.* Number; *FIGO* International Federation of Gynecology and Obstetrics.

^#^Pearson chi-square test.

### Prognostic significance of LGR5 in high grade serous carcinomas

**Figure 6:**
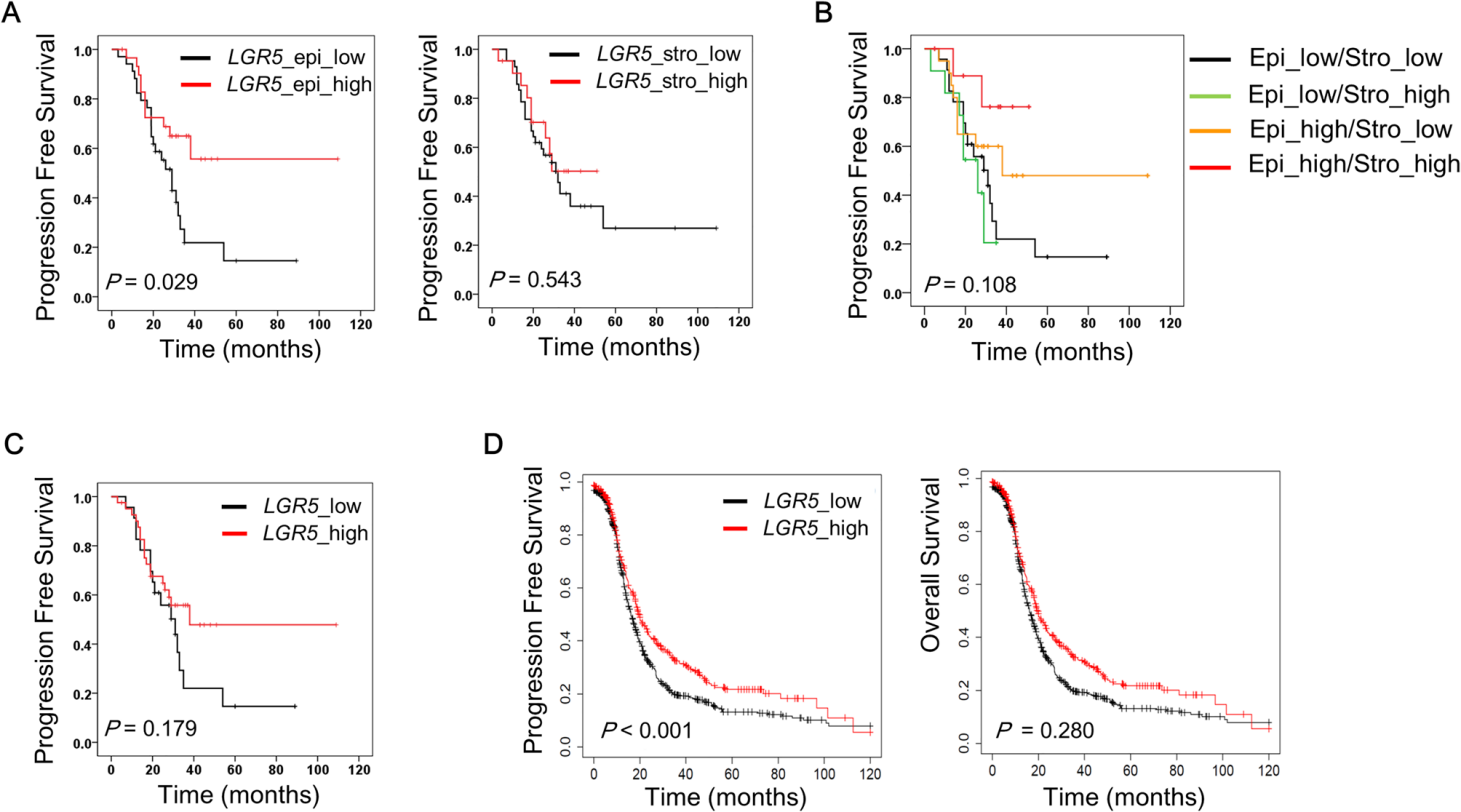
Prognostic value of *LGR5* expression in ovarian serous carcinoma patients. *LGR5* expression was classified into low and high according to the histo-scores of *LGR5* (cutoff value: 40) in carcinoma cells or stromal cells in HGSC. (n = 64) (**A**) High *LGR5* expression in carcinoma cells is significantly associated with better progression free survival (PFS) in HGSC patients, whereas stromal *LGR5* expression is not associated with PFS. (**B**) Progression free survival with respect to epithelial and stromal *LGR5* expression. (**C**) Progression free survival in global *LGR5-*low and-high groups. (**D**) An independent survival analysis was performed using Kaplan–Meier plotter, an online database, on ovarian serous carcinoma patients (Affy ID: 213880_at, follow up: 120 months, n = 1104). The optimal cutoff value for low- and high-LGR5 expression was automatically selected. *LGR5*-high serous carcinomas had better PFS rates (*P* < 0.001, hazard ratio: 0.76). However, *LGR5* expression had no impact on OS.

As stromal *LGR5* expression was observed in 33% of HGSC cases, including 11 cases of stroma only *LGR5* expression, we separately evaluated the prognostic impact of epithelial or stromal *LGR5* expression in HGSC patients (n = 64). Survival analysis demonstrated that high *LGR5* expression in epithelial tumor cells correlated with improved progression free survival (PFS) rates (*P* = 0.029) (Fig. 6A). In contrast, stromal *LGR5* expression had no influence on PFS (*P* = 0.543) (Fig. 6A). Remarkably, multivariate analysis demonstrated that epithelial *LGR5* expression (*P* = 0.018) is an independent prognostic marker along with FIGO stage (*P* = 0.033) and the presence of residual tumor after operation (*P* = 0.047) (Table 2). Next, we classified HGSC patients into 4 groups based on the epithelial and stromal expression of *LGR5*: epithelial (Epi)-high/stromal (Stro)-high, Epi-high/Stro-low, Epi-low/Stro-high, and Epi-low/Stro-low. Interestingly, the Stro-high group showed relatively better PFS than the Stro-low group when epithelial *LGR5* expression was high even though the case number was too small to make a definitive conclusion (Fig. 6B). When cases were classified into global *LGR5*-low (Epi-low /Stro-low) and -high (either Epi-high or Stro-high) groups by combining epithelial and stromal *LGR5* expression, *LGR5*-high group showed better prognostic significance although it did not reach the statistical significance (*P* = 0.179) (Fig. 6C). To verify the prognostic impact of *LGR5*, we performed additional survival analysis with an independent cohort using Kaplan–Meier plotter for ovarian serous carcinomas (n = 1104), an online tool for the validation of prognostic biomarker candidates based on transcriptome data^28^. Although this cohort was not restricted to HGSC cases, the Kaplan–Meier curves showed results consistent with our findings; high *LGR5* expression was significantly associated with better PFS rates (*P* < 0.001), whereas no significant difference was observed in OS (*P* = 0.280) (Fig. 6D).

**Table 2:** The univariate and multivariate analysis for progression free survival rate for high-grade serous carcinomas.

Variables		Univariate analysis		Multivariate analysis	
		HR (95% CI)	*P-*value	HR (95% CI)	*P-*value^a^
Age	< 55/ ≥ 55	0.908 (0.461–1.788)	0.780		
Family history of ovarian cancer	Absent/present	0.892 (0.541–1.470)	0.653		
Menopause	No/Yes	1.029 (0.515–2.057)	0.935		
Serum CA-125	< 700/ ≥ 700	0.298 (0.726–2.841)	0.298		
FIGO stage	1–2/3/4	1.995 (1.127–3.532)	0.018	1.913 (1.054–3.473)	0.033
Malignant ascites	Absent/present	0.816 (0.427–1.559)	0.538		
Residual tumor after operation	Absent/present	2.271 (1.146–4.500)	0.019	2.043 (1.010–4.129)	0.047
Germline BRCA1 or 2 mutations	Absent/present	0.543 (0.267–1.1.5)	0.092		
Platinum sensitivity	Sensitive/resistant	2.149 (0.882–5.236)	0.092		
Estrogen receptor	Negative/positive	0.802 (0.391–1.645)	0.547		
Stromal LGR5	Low/high	0.790 (0.367–1.703)	0.548		
Epithelial LGR5	Low/high	0.456 (0.220–0.944)	0.034	0.412 (0.198–0.856)	0.018

*HR* Hazard ratio; *CI* confidence interval; *FIGO* International Federation of Gynecology and Obstetrics.

^a^Cox proportional hazard model.

**Figure 7:**
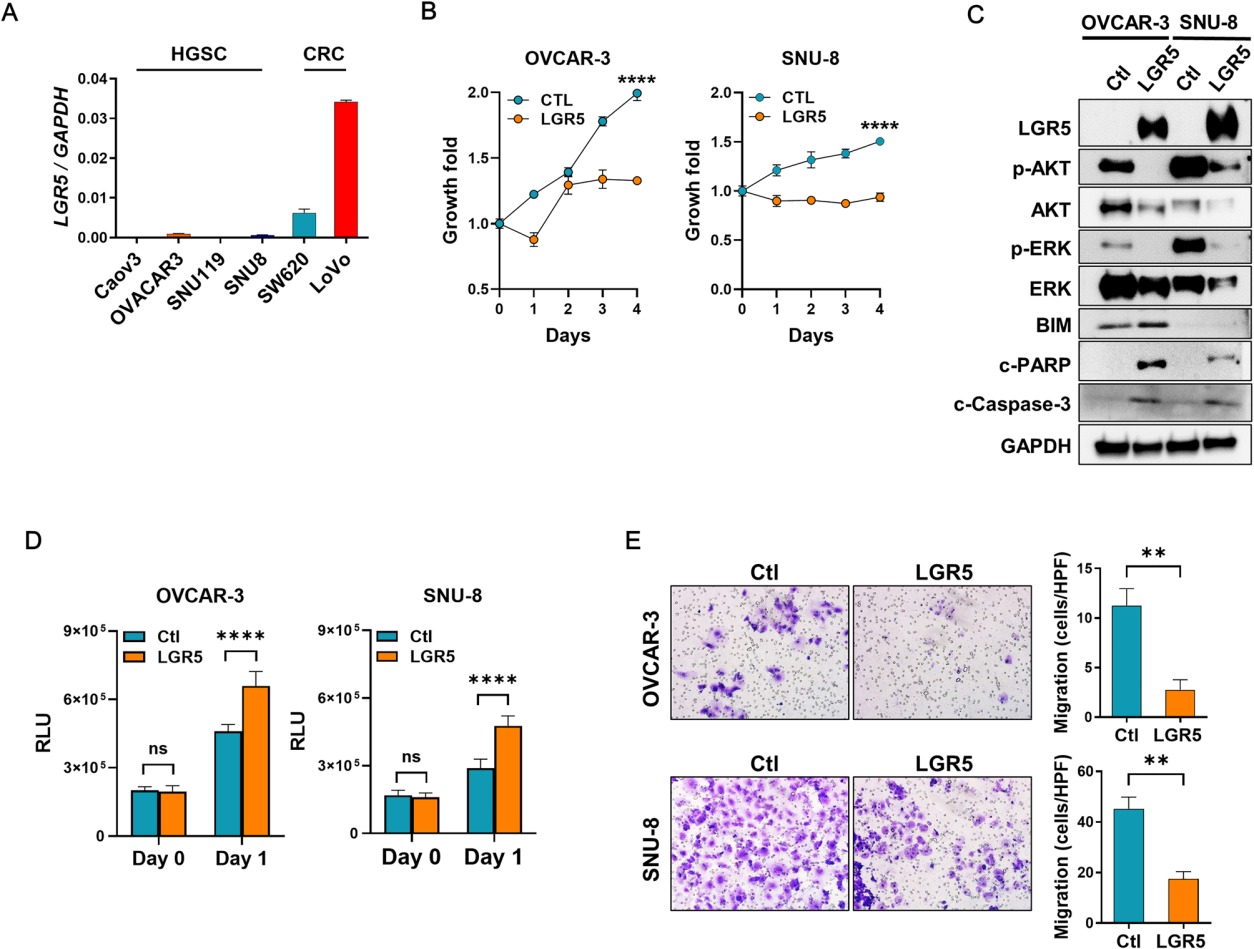
The suppressive effects of LGR5 on high-grade serous carcinoma (HGSC) cell growth and migration. (**A**) Real-time PCR was performed to measure the mRNA levels of *LGR5* in four human HGSC cell lines compared to colorectal cancer (CRC) cell lines, SW620 and LoVo. (**B**) OVAR-3 and SNU-8 cells were transfected with a control or a LGR5-expressing plasmid. Cell growth was measured using a Cell Counting Kit-8 at the indicated times. (**C**) Twenty-four h after transfection with a control or a LGR5-expressing plasmid in OVCAR-3 and SNU-8 cells, an immunoblot assay for survival and apoptosis-related proteins was performed using the antibodies indicated in the figure. (**D**) Caspase-3 activity was measured after transfecting cells with a control or a LGR5-expressing plasmid. (**E**) The effect of LGR5 expression on the migration activity of OVCAR-3 and SNU-8 cells was evaluated using a transwell migration assay. Cellular migration was imaged at 0 and 48 h. *c-PARP* cleaved PARP; *c-caspase-3* cleaved caspase-3; *Ctl* control; *RLU* relative light unit. Data are presented as the mean ± SD. **P < 0.01, ****P < 0.0001 by unpaired t test.

To explore the functional roles of LGR5 in HGSC, we screened 4 HGSC cell lines and found that *LGR5* mRNA levels were extremely low in all of them compared to the colon cancer cell lines, LoVo and SW620 (Fig. 7A). To determine the effect of LGR5 on tumor growth, we induced LGR5 expression in OVCAR-3 and SNU-8 cells. We observed significantly reduced proliferation rates in cancer cells transfected with LGR5 compared to those transfected with a control plasmid (Fig. 7B). As AKT or MAPK signaling pathways are often involved in cancer cell survival, we examined the activation of AKT and ERK proteins and found that LGR5 overexpression resulted in the downregulation of AKT and ERK phosphorylation as well as AKT and ERK protein levels (Fig. 7C). Additionally, we examined apoptosis-related molecules. LGR5 overexpression increased BIM expression in OVCAR-3 cells, but not in SNU-8 cells (Fig. 7C). Cleaved PARP and cleaved caspase-3 also increased in LGR5-transfected tumor cells (Fig. 7C). In line with these findings, the caspase-3 activity assay confirmed that LGR5 expression induced increased apoptosis in both cell lines (Fig. 7D). Furthermore, decreased migratory activity was observed in the transwell migration assays in response to LGR5 transfection in OVCAR-3 and SNU-8 cells (Fig. 7E). Taken together, our results suggest that LGR5 may act as a tumor suppressor in HGSC progression.

### *LGR5* expression in nonserous ovarian carcinomas

**Figure 8:**
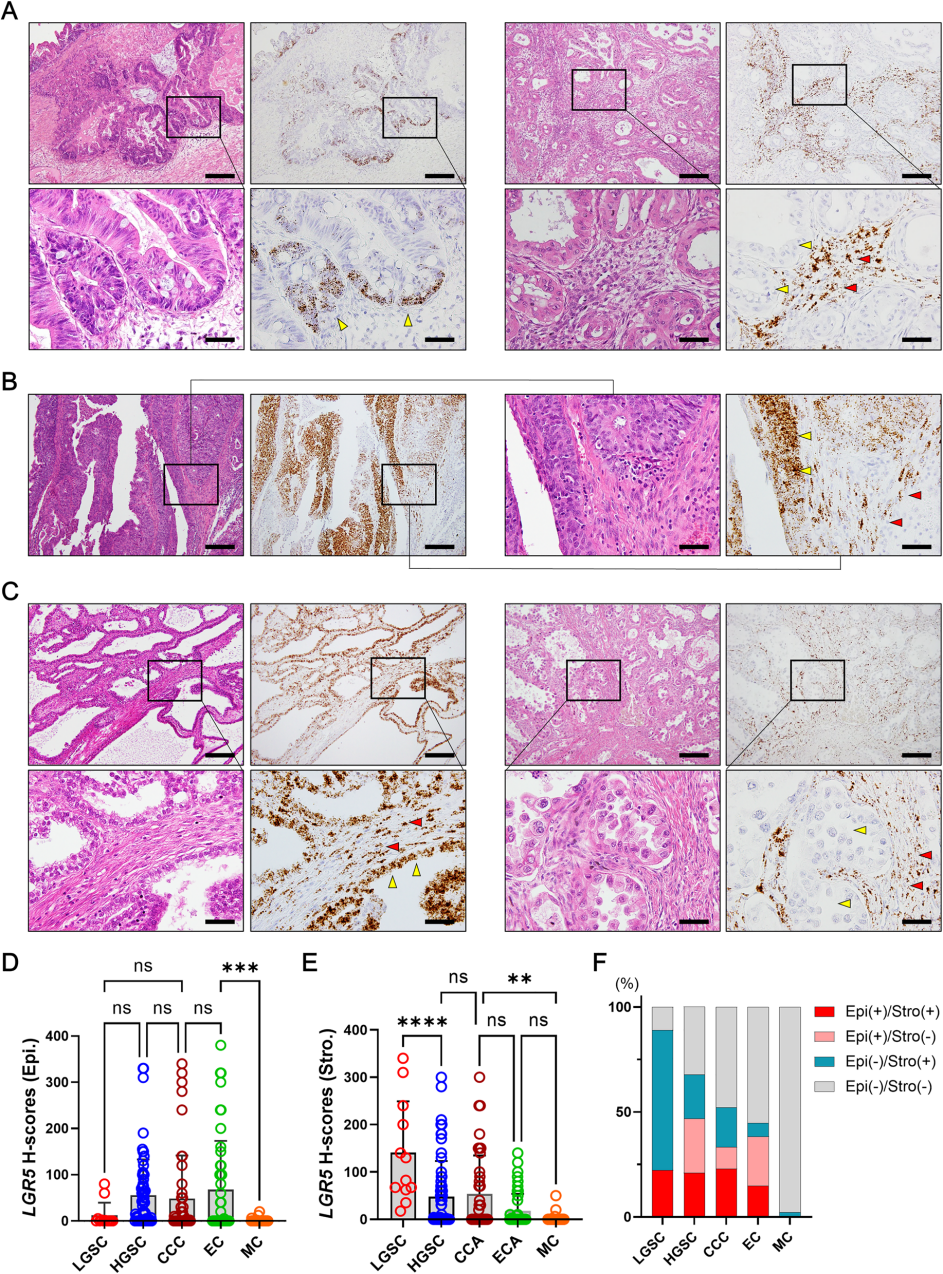
*LGR5* expression in various histological subtypes of ovarian carcinomas. Representative images of *LGR5* expression in mucinous carcinoma (MC, n = 44) (**A**), endometrioid carcinoma (EC, n = 47) (**B**), and clear cell carcinomas (CCC, n = 48) (**C**). Scatter plots showing histo-scores (H-scores) of epithelial (**D**) or stromal (**E**) *LGR5* expression in each subtype of ovarian carcinoma. (**F**) A bar graph showing the proportions of epithelial and stromal *LGR5* positivity in each subtype of ovarian carcinomas with an H-score cutoff value of 40. Low-grade serous carcinoma (LGSC, n = 12), High-grade serous carcinoma (HGSC, n = 64). Scale bar, 0.2 mm (left two panels) and 50 µm (right two panels) (**A**,**C**); 0.2 mm (upper panels) and 50 µm (lower panels) (**B**). *ns* not significant. **P < 0.01, ***P < 0.001, ****P < 0.0001 by Tukey’s multiple comparisons test.

Next, we investigated *LGR5* expression in other histologic types of ovarian carcinomas, including 44 mucinous carcinomas (MCs), 47 endometrioid carcinomas (ECs), and 48 clear cell carcinomas (CCCs). The majority of MCs were negative for *LGR5*, and only a few cases displayed *LGR5* expression (H-scores [mean ± SD]: 9.2 ± 49.6, Fig. 8A). Mucinous cystadenomas also expressed negligible levels of *LGR5* (mean ± SD: 1.1 ± 2.6), whereas mucinous borderline tumors showed lightly higher levels of *LGR5* (mean ± SD: 13.5 ± 17.7) (Supplementary Fig. 6). A significant number of ECs highly expressed *LGR5* (mean ± SD: 68.0 ± 105.1, Fig. 8B) and there were many cases of CCCs that were strongly positive for *LGR5* in stromal cells with or without epithelial *LGR5* expression (mean ± SD: 48.5 ± 92.9, Fig. 8C). Comparing *LGR5* H-scores in carcinoma cells, LGSC and MC expressed much lower levels of *LGR5* than other subtypes (Fig. 8D). On the other hand, LGSC exhibited the highest levels of stromal *LGR5* expression (mean ± SD: 89.2 ± 57.3), while it was much lower in ECs (mean ± SD: 17.9 ± 36.1) and MCs (mean ± SD: 13.7 ± 48.1) (Fig. 8E). The proportion of epithelial and/or stromal positivity (cutoff value: 40 of H-score) varied between subtypes (Fig. 8F), and we suggest three categories of ovarian carcinomas based on *LGR5* expression; stromal-predominant type (LGSC), epithelial-stromal type (HGSC, CCC, EC), and low type (MC).

### Nuclear β-catenin expression in ovarian carcinomas and its correlation with *LGR5*

**Figure 9:**
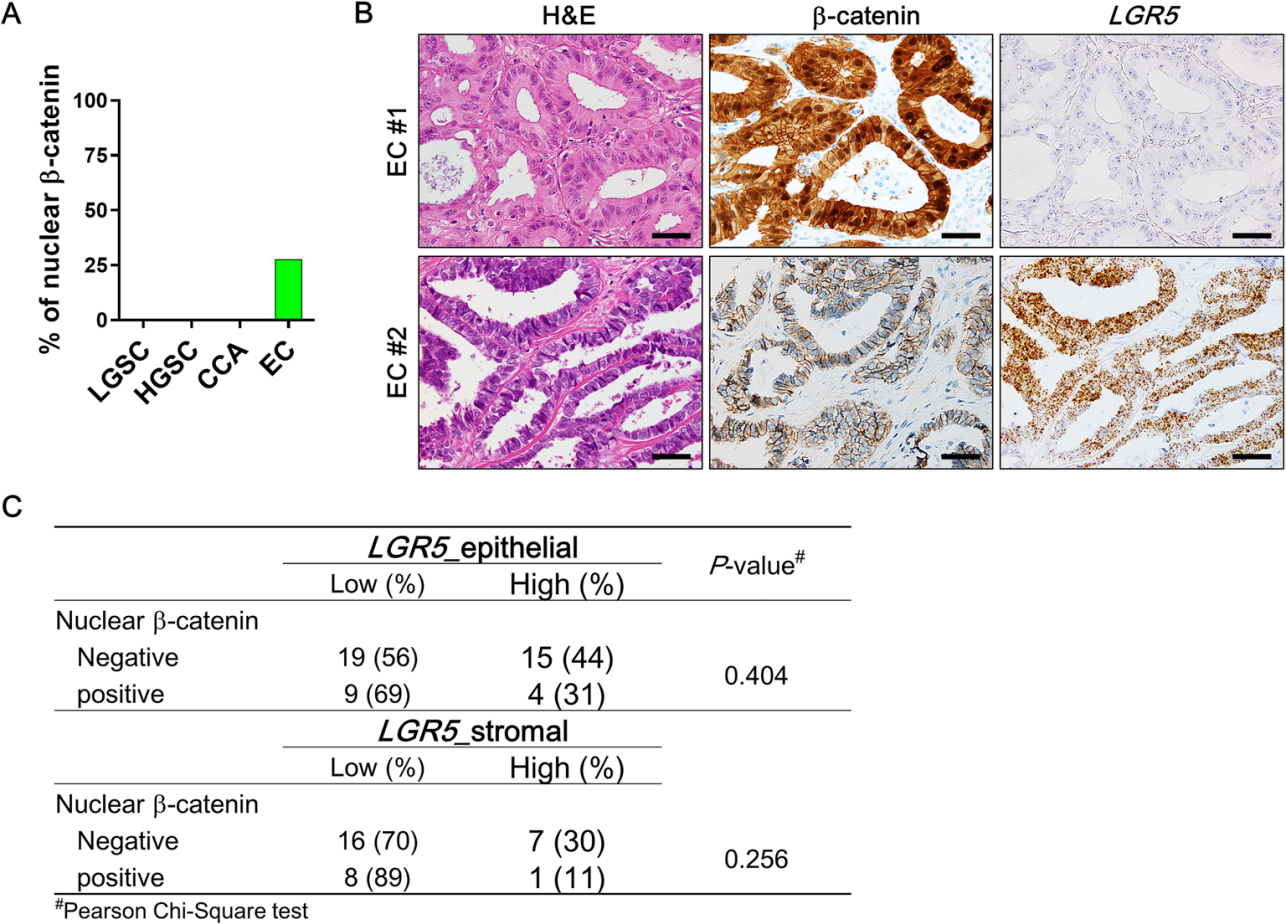
Association of *LGR5* with β-catenin expression in endometrioid carcinomas (ECs). (**A**) A bar graph showing percentages of nuclear β-catenin expression in various ovarian carcinomas. (**B**) Representative images of a EC expressing nuclear β-catenin and no *LGR5* (EC #1), and a EC expressing normal membranous β-catenin and strong *LGR5* (EC #2). Scale bar, 50 µm (**C**) A table showing the correlations of epithelial or stromal *LGR5* with nuclear β-catenin expression in ECs. LGSC, low-grade serous carcinoma (n = 12); HGSC, high-grade serous carcinoma (n = 64); CCA, clear cell carcinoma (n = 48).

*LGR5* is one of the Wnt target genes, and its overexpression has been associated with abnormally enhanced Wnt/β-catenin signaling in many types of cancers. Thus, we examined whether Wnt signaling activity is responsible for the high levels of *LGR5* expression observed in ovarian carcinomas by examining the correlation between nuclear β-catenin and *LGR5* expression. Notably, nuclear β-catenin expression was only detected in EC (27%, 13 of 47 cases) but not in other histological subtypes such as LGSC, HGSC, and CCA (Fig. 9A). Furthermore, nuclear β-catenin in EC showed no association with epithelial or stromal *LGR5* expression (Fig. 9B,C). These findings clearly indicate that Wnt/β-catenin pathway is not involved in the regulation of *LGR5* expression in ovarian carcinomas.

## Discussion

**Figure 10:**
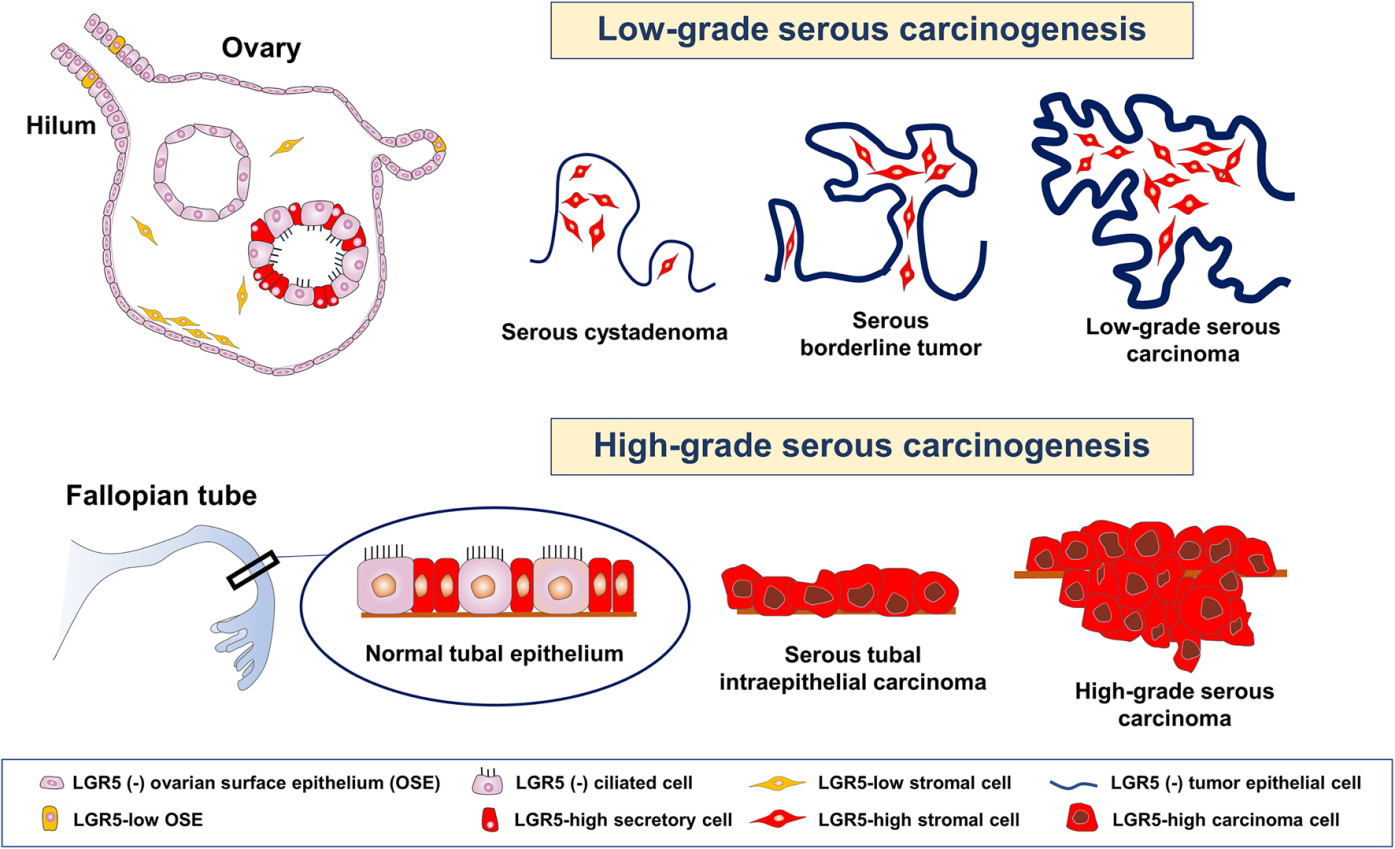
Schematic representation of distribution of *LGR5* expression in ovary, fallopian tube, and ovarian serous carcinogenesis.

Lgr5 has been identified as a promising stem cell marker in multiple organs of adult mice. Lineage tracing convincingly demonstrated that Lgr5^+^ cells are stem/progenitor cells in the murine ovary and oviduct^19^. In this study, we thoroughly investigated *LGR5* expression in human ovaries and fallopian tubes as well as related benign and malignant ovarian tumors. Importantly, we discovered that *LGR5* is highly expressed in the normal fallopian tube epithelium and that *LGR5* expression in ICs and serous cystadenomas is closely associated with a Müllerian phenotype. By analyzing the *LGR5* expression profile, we demonstrated that strong stromal *LGR5* expression is characteristic of type 1 ovarian carcinogenesis (Fig. 10). Furthermore, our survival analyses and *in vitr*o assays suggested that *LGR5* plays tumor suppressive roles in HGSC.

In mice, Lgr5^+^ cells are present in the OSE and hilum and expand in response to tissue injury upon ovulation^19,20^. Although we observed a few *LGR5*^+^ cells in the hilar area and in the proliferative OSE in the human ovary, we did not observe any *LGR5* expression in homeostatic OSE. This discrepancy can be probably due to inherent biological differences between species or it may be derived from differences in the technique used to detect *LGR5* expression, a reporter gene in a lineage tracing vs. RNA ISH. Additionally, unlike mouse studies in which ovulation can be induced and the response of OSE to tissue injury can be examined, there is no way to obtain human ovary samples that have just undergone ovulation. In fact, regarding *LGR5* expression, we recently reported a similar discrepancy in the breast between mouse and human tissues. Lgr5^+^ cells have been identified in the proximal ducts as resident stem cells in the murine mammary glands^11,29,30^, but we did not detect any *LGR5* expression in the human mammary ducts. We only observed a small number of LGR5^+^ cells in the regenerating ducts^31^. In general, RNA ISH seems to be a sensitive and specific tool for detecting *LGR5* expression in human FFPE tissues; however, if its expression is extremely weak or temporary in certain organs, caution should be taken when interpreting the results to avoid false negatives. Indeed, *Lgr5* has been demonstrated to be a facultative stem cell marker in the murine liver, pancreas, and stomach^13,14,32^, indicating that *Lgr5*^+^ cells may emerge only in response to tissue injury for regeneration.

In contrast to the rare and weak expression of *LGR5* in OSE, it was remarkable to find strong *LGR* expression in ICs and which was exceptionally observed in ICs with tubal (or Müllerian) metaplasia. This relationship between *LGR5* and tubal metaplasia was also observed in SCs; SCs with TE exhibited much higher *LGR5* expression than those with NTE. This may be explained by the fact that their epithelial linings are almost identical. In mice, Lgr5^+^ cells in Müllerian ducts were identified as embryonic stem/progenitor populations contributing to the development of the epithelia of the adult oviduct^19,33^. These findings suggest that *LGR5*^+^ cells can reappear when Müllerian metaplasia occurs in the benign cystic lesions of adult human ovaries. An increased *LGR5*^+^ cell population in the metaplastic process was also reported during intestinal metaplasia in the human stomach, and *LGR5*^+^ cells were suggested to be the cell of origin of gastric tumors^34^. Considering that some HGSCs are believed to arise potentially from these ICs with a Müllerian phenotype^35,36^ and Müllerian metaplasia is accompanied by *LGR5*^+^ cell expansion, it is possible that these *LGR5*^+^ cells might contribute to tumor development.

Here, we clearly demonstrated that *LGR5* was highly expressed in the normal fallopian tube epithelium, which is well consistent with the previous reports in which RT–PCR and ISH were employed to determine the presence of *LGR5*^+^ cells^19,37^. We further revealed that not only the distal fallopian tube/fimbria but also the ampulla region displays strong *LGR5* expression. The fallopian tube mucosa contains columnar epithelium that consists of two main cells: ciliated cells and secretory cells. Ciliated cells function to transport an oocyte from the ovary toward the uterus, and secretory cells contain apical granules and produce tubal fluid. More interestingly, we discovered that *LGR5* expression is strictly confined to secretory cells. By combining RNA ISH for *LGR5* and multiple IHC for FOXJ1 and PAX8, we demonstrated specific *LGR5* expression only in the PAX8 + /FOXJ1- secretory cells. STIC and HGSCs have been shown to originate in PAX8^+^ secretory cells by targeting the Brca, Tp53, and Pten genes^38^. More recently, Yamamoto et al. reported that established clones of fallopian tube stem cells that can propagate through multiple passages exhibit strong and consistent staining of PAX8, indicating a secretory cell lineage^39^. Along with these findings, our results of exceptional *LGR5* expression in the secretory cells may support the notion that secretory cells harbor a group of stem cell populations in the fallopian tube, acting as cells of origin for this cancer. However, it seems unlikely that all *LGR5*^+^ cells in the fallopian tube are stem cells because the number of *LGR5*^+^ cells is too high; nearly all secretory cells are positive for *LGR5*. Therefore, unlike in other organs such as the stomach, intestine, and hair follicle, *LGR5*^+^ cells in the fallopian tube may contain a much wider range of cell populations, for example stem, progenitor cells, and even differentiated cells.

One of the most remarkable findings in this study was that human ovarian stromal cells express *LGR5*, in contrast to the results from mouse studies in which ovarian stromal cells in adult mice express no *Lgr5*^19^. In most normal human ovaries examined, *LGR5*^+^ cells were observed throughout the stroma although the percentage of positive cells and their expression intensity were low. Stromal *LGR5* expression was often accentuated immediately beneath the ovarian surface, and notably, this subepithelial *LGR5* expression was predominantly observed during the low-grade serous carcinogenesis pathway; five of 15 SAs showed subepithelial *LGR5* expression, and the majority of SBTs and LGSCs displayed high levels of *LGR5* expression in stromal cells, particularly those adjacent to epithelial tumor cells. Histologically, SBT and LGSC are characterized by thick and bulbous papillae filled with an abundant amount of stroma. An increased population of *LGR5*-positive stromal cells might be responsible for the formation of this characteristic structure. Interestingly, we observed an SC lined by NTE that displayed stromal *LGR5* as strong as SBT and LGSC (Fig. 2). This finding led us to hypothesize that an SC with strong subepithelial *LGR5* expression might represent a true precursor that has the potential to progress to SBT and LGSC. In addition, we observed the possibility that SC with TE can progress to SBT because we observed an SBT expressing *LGR5* both in epithelial and stromal cells even though the incidence was low (14%, 1 of 7 cases). Taken together, we believe that this stroma-predominant expression pattern of *LGR5* during low-grade serous carcinogenesis could be additional evidence that supports the concept of the type I pathway along with distinct histologic and molecular characteristics.

In our previous studies that investigated *LGR5* expression in carcinomas of the stomach, colorectum, and breast, we observed no *LGR5* expression in cancer-associated fibroblasts (CAFs) even in cases accompanied by extensive stromal reactions^24,31,40^. In contrast, significant levels of stromal *LGR5* expression were observed in all histological types of ovarian carcinomas. We determined that this striking difference may be due to the unique histological component of the ovary, ovarian stroma. Considering that CAFs mostly originate from local stromal cells, ovarian CAFs likely arise from ovarian stromal cells. Indeed, by analyzing the expression of FOXL2, a marker specific to ovarian stromal cells, Fugisawa et al. have demonstrated that ovarian stromal cells are the primary source of ovarian cancer stroma^41^. Given that a small number of *LGR5*^+^ stromal cells are normally present in the subepithelial space and deep ovarian stroma as we demonstrated in this study, it might be reasonable to speculate that those rare *LGR5*^+^ stromal cells are induced to proliferate by tumor cells and contribute to the *LGR5*-rich cancer stroma.

The ovarian stroma has been proposed to play an important role in ovarian cancer development and progression. Blanco Jr et al. suggested potential interplay between the tumors and the ovarian stroma; the stroma surrounding epithelial tumors in the ovary can be activated to release steroid hormones which may stimulate further neoplastic growth^42^. It has been described in mice that subsurface *Lgr5* + cells are observed at embryonic day 13.5 and postnatal day1, and then disappear as of postnatal day 7^19^. Therefore, this coupled epithelial-stromal expression of *LGR5* in ovarian tumors might recapitulate the expression pattern observed during the early developmental stage. Importantly, the functional implication of *Lgr5*^+^ stromal cells has also been reported in the bronchus of the lung; Lee et al. demonstrated that *Lgr5* and *Lgr6* are markers of mesenchymal cells in the adult lung, where distinct *Lgr5*^+^ cells are located in alveolar compartments and are necessary to promote alveolar differentiation of epithelial progenitors through Wnt activation^43^. Therefore, it is possible that there are specific cellular partnerships between *LGR5*^+^ stromal cells and *LGR5*^+^ or *LGR5*^−^ tumor cells. The precise mechanisms by which *LGR5* expression is regulated in stromal and epithelial cells and its functional significance in the interaction between these cell types require further investigation.

The prognostic significance of *LGR5* has been investigated in various types of carcinomas; however, the results remain controversial depending on the tumor type^44^. It seems that ovarian carcinomas are not an exception. Sun et al. have evaluated LGR5 expression by IHC and found that it is associated with advanced stages, higher grades, and poor overall survival^45^. Liu et al. also reported that suppression of LGR5 expression led to decreased proliferation and metastasis, suggesting a role in the progression of ovarian cancers^46^. However, as mentioned previously, IHC analysis for LGR5 on FFPE specimens is not reliable. Here, even though the number of cases was not sufficient to make a definitive conclusion, we found that epithelial *LGR5* expression is associated with improved PFS in HGSC patients and we observed consistent results in an independent cohort with a large number of serous carcinoma patients using an online database. *LGR5* is highly expressed in the secretory cells of normal tubal epithelium, and its expression is decreased in HGSCs compared to precursor STIC lesions. Similarly, in a mouse endometrial cancer model, it has been shown that *Lgr5* is highly expressed in the epithelium during the initial stages of tumorigenesis but is remarkably down-regulated in fully developed tumors^47^. Moreover, we demonstrated the inhibitory effects of LGR5 expression on tumor growth and migration abilities using two HGSC cell lines. This is opposite to the previous result mentioned above^46^, it might be probably due to the difference in the cell lines used for functional studies. In the study of Liu et al., SKOV3 was identified to express high level of LGR5 and used to investigate the functional role of LGR5 by suppressing its expression. However, there have been serious doubts about the histologic identity of SKOV3, and several studies demonstrated that, according to genetic profiling, SKOV3 is classified as “unlikely HGSC” and is more likely to be endometrioid or clear cell carcinoma^48–51^. On the other hand, OVCAR-3 and SNU-8 cell lines used in this study were classified as “possibly HGSC”^48^. Although our findings suggest that *LGR5* may play suppressive roles in the progression of ovarian HGSC, more thorough functional studies are needed to draw definitive conclusions as we have not provided an effect of LGR5 downregulation in HGSC cell lines. In addition, further studies with a larger size of samples are also required to confirm the prognostic significance of *LGR5* not only in HGSC but also in other types of ovary carcinomas.

*LGR5* was initially identified as a Wnt target gene, and its expression is closely associated with dysregulation of the Wnt/β-catenin pathway in cancers. For example, *LGR5* expression is frequently observed in hepatocellular carcinoma with β-catenin mutations^52^, and in colorectal cancers, it is positively correlated with nuclear β-catenin expression^24^. Although gene mutations in the Wnt/β-catenin pathway are relatively uncommon in ovarian cancer in general, in this study, we observed high levels of *LGR5* expression in all subtypes of ovarian carcinomas, except MCs. Therefore, we investigated whether this increased *LGR5* expression is related to enhanced Wnt signaling activity. However, nuclear β-catenin expression was only detected in ECs (27%, 13 of 47 cases) but not in other histological subtypes, which appears to be consistent with previous studies reporting 16 to 54% of β-catenin mutations in ECs^53^. Moreover, it was unexpected to find that nuclear β-catenin in ECs had no association with *LGR5* expression. These results suggest that the Wnt/β-catenin signaling is less likely to be responsible for *LGR5* expression in ovarian carcinomas. Interestingly, it has recently been demonstrated that nuclear localization of β-catenin is not observed in any breast cancers expressing *LGR5*^31^. Therefore, it seems that *LGR5* expression can be regulated by signaling pathways other than Wnt/β-catenin depending on the type of cancer.

In summary, we discovered that *LGR5* is rarely expressed in the ovary surface epithelium but is specifically and highly expressed in the secretory cells of fallopian tube epithelium as well as in the tubal metaplasia in ICs. In the LGSC pathway, *LGR5* displays a distinct stroma-predominant expression pattern, whereas it displays various epithelial-stromal expression in HGSC, CCC, and EC. In particular, *LGR5* expression declines throughout the progression from STIC to invasive carcinoma and is significantly associated with improved PFS in HGSC patients. In addition, LGR5 overexpression results in decreased tumor growth and migratory abilities in HGSC cell lines. These findings suggest tumor-suppressive roles for *LGR5* in high-grade serous carcinogenesis in the ovary.

## Supplementary Information

Supplementary Information.

## Data Availability

The datasets used and/or analyzed during the current study are available from the corresponding author upon reasonable request.
