# Supplementary material for: Differential epithelial and stromal LGR5 expression in ovarian carcinogenesis
Source: Sci Rep. 2022 Jul 1;12:11200. doi: 10.1038/s41598-022-15234-2 (PMC9249864; doi:10.1038/s41598-022-15234-2)

Differential epithelial and stromal *LGR5* expression in ovarian carcinogenesis

Hyesung Kim[1], Dong Hui Lee[1], Eunsun Park[1], Jae Kyung Myung[2], Jeong Hwan Park[3], Dong Il Kim[4], Se Ik Kim[5], Maria Lee[5], Younghoon Kim[6], Chul Min Park[7], Cheol Lee[8], Bogun Jang[1]

[1]Department of Pathology, Jeju National University School of Medicine, Jeju, South Korea

[2]Department of Pathology, Hanyang University College of Medicine, Seoul, South Korea

[3]Department of Pathology, SMG-SNU Boramae Medical Center, Seoul, South Korea

[4]Department of Pathology, Green Cross Laboratories, Yongin, Gyeonggi, South Korea.

[5]Department of Obstetrics and Gynecology, Seoul National University College of Medicine, Seoul, South Korea

[6]Laboratory of Epigenetics, Cancer Research Institute, Seoul National University College of Medicine, Seoul, South Korea

[7]Department of Obstetrics and Gynecology, Jeju National University School of Medicine, Jeju, South Korea

[8]Department of Pathology, Seoul National University College of Medicine, Seoul, South Korea

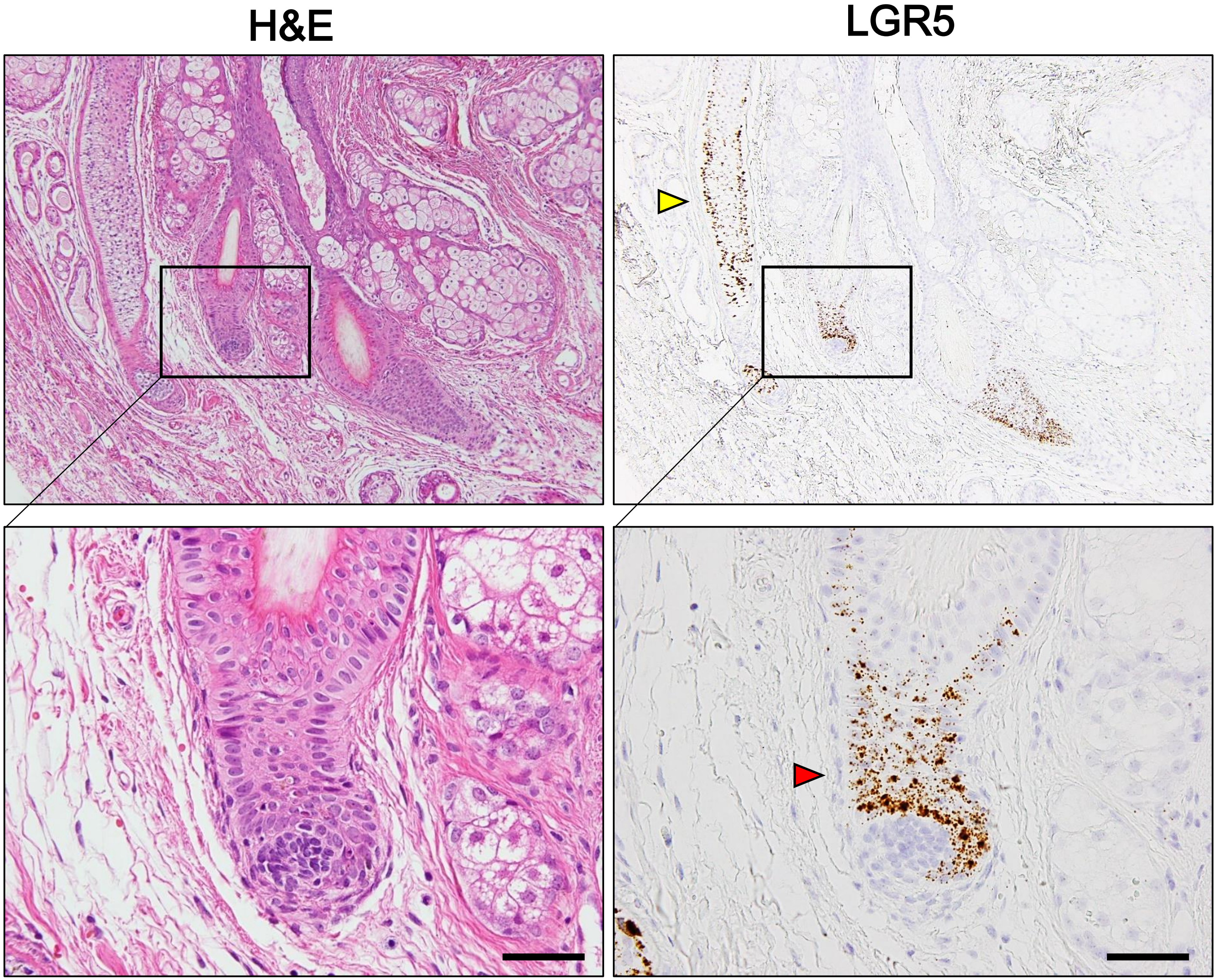

Supplementary Figure 1 *LGR5* expression in hair follicles observed in mature cystic teratoma of the ovary. The tumor is mostly composed of stratified squamous epithelium and skin appendages including hair follicles, sebaceous glands and sweat glands. *LGR5* expression is specifically detected in outer root sheath (indicated by yellow arrowhead) and bulb (indicated by red arrowhead) areas. Scale bar: 50μm

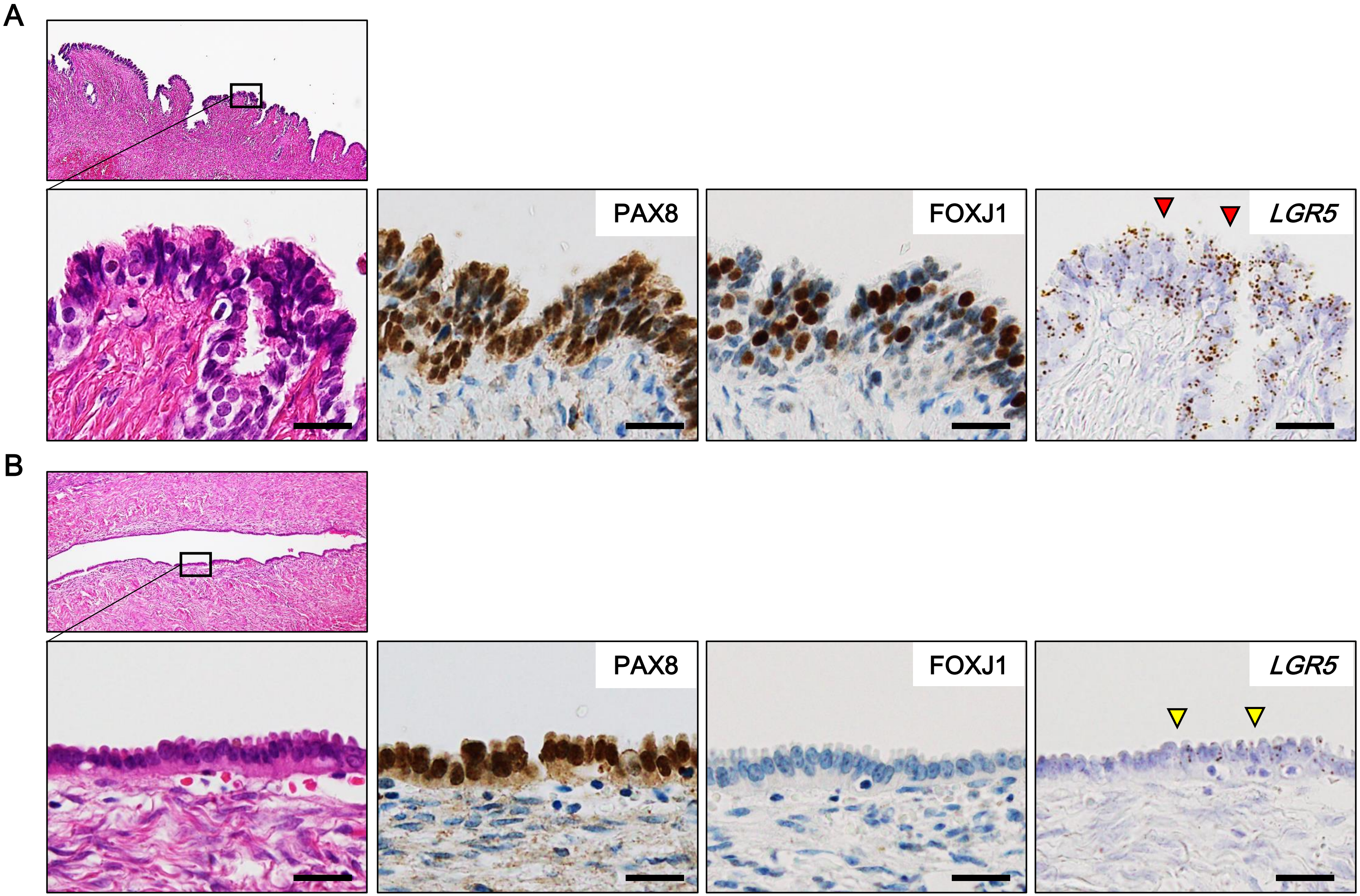

Supplementary Figure 2 *LGR5* expression in serous cystadenoma lined by tubal type or nontubal type epithelium. (A) Tubal type epithelium of serous cystadenoma, comprised of secretory cells positive for PAX8 and ciliated cells expressing FOXJ1, expresses high levels of *LGR5* (indicated by red arrowheads). Scale bar, 25 μm. (**B)** In serous cystadenoma lined by nontubal type epithelium, epithelial cells are positive for PAX8 but negative for FOXJ1. Tumor epithelial cells express no or very low levels of *LGR5* (indicated by yellow arrowheads). Scale bar, 25 μm.

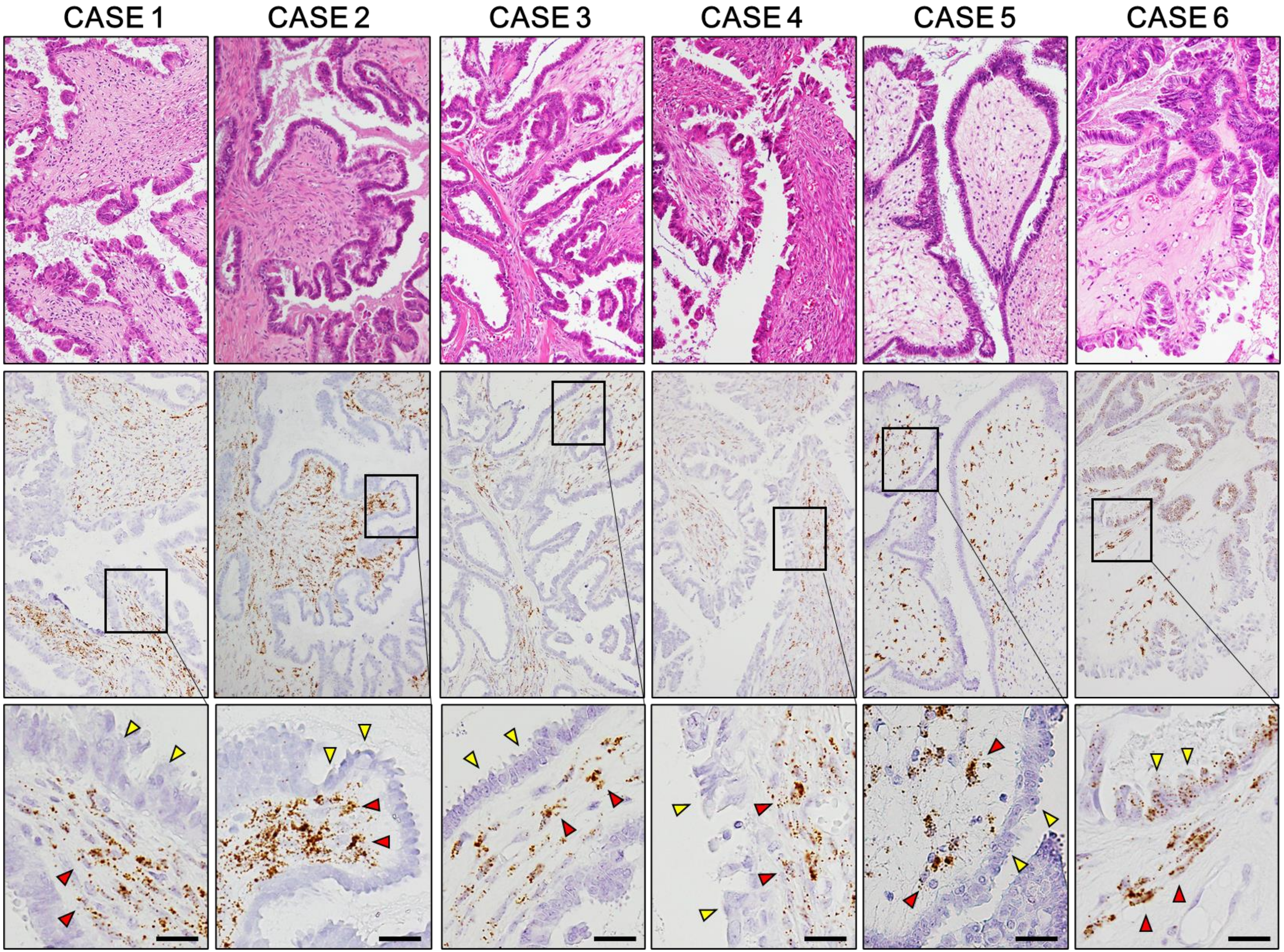

Supplementary Figure 3 *LGR5* expression in serous borderline tumors. Seven cases of serous borderline tumors were examined. In 6 of them (case 1, 2, 3, 4, 5 and 7), *LGR5* was exclusively expressed in the tumor stromal cells. Case 7 is shown in Figure 3. In case 6, *LGR5* expression was observed in both epithelial cells and stromal cells. Yellow arrowheads indicate epithelial tumor cells and red arrowheads indicate tumor stromal cells. Scale bar, 25 μm

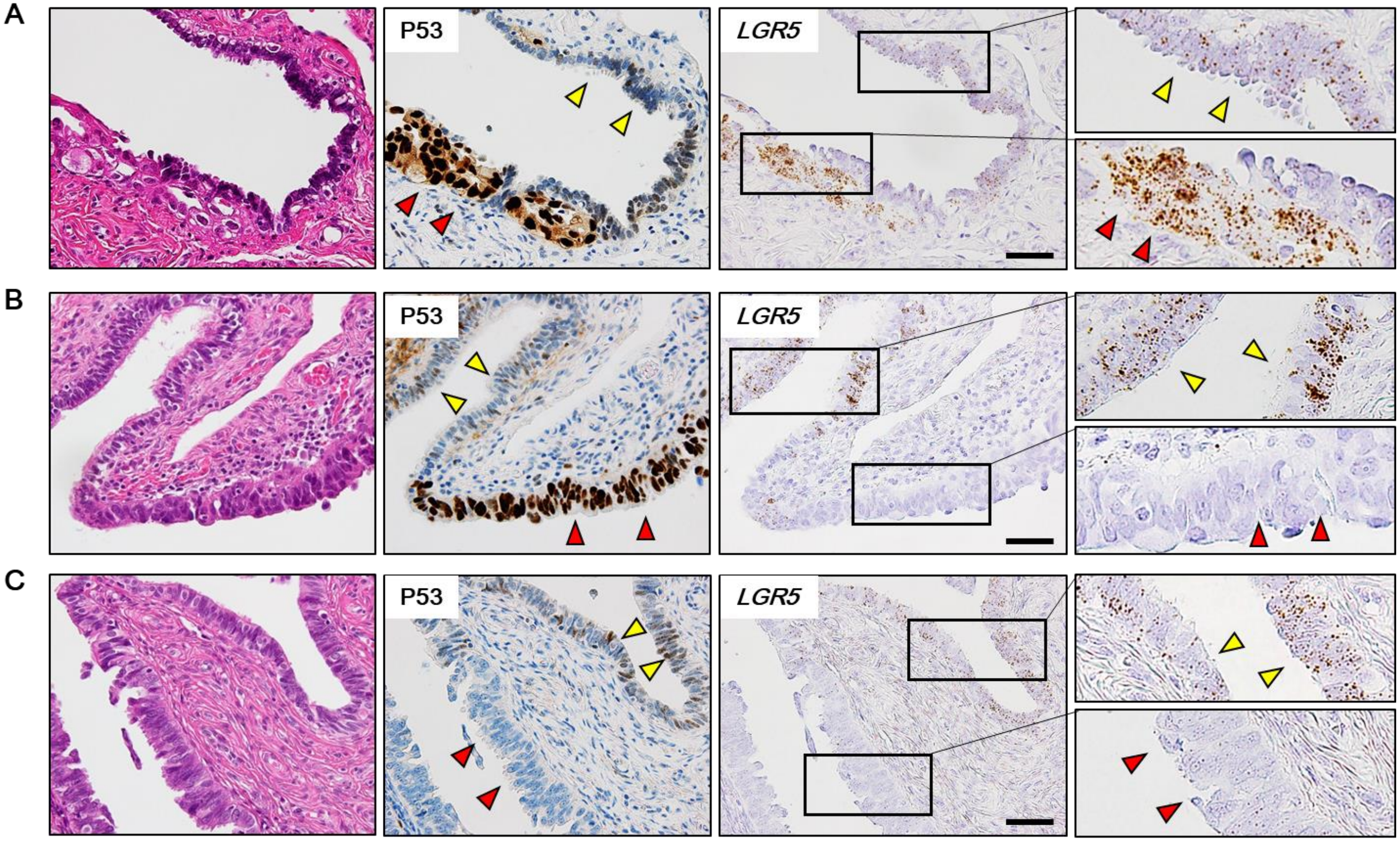

D

Histo-scores of *LGR5* in serous tubal intraepithelial carcinoma and adjacent non-tumorus tubal epithelium.

No.	1	2	3	4	5	6	7	8	9	10	11	12	13	14	15	16	17	18	19	20	21
NTE	93	75	180	100	80	30	40	31	70	110	140	60	40	10	150	200	180	40	55	30	20
STIC	97	70	30	50	20	160	150	135	100	40	0	245	60	50	240	220	90	150	70	0	195

Supplementary Figure 4 *LGR5* expression in serous tubal intraepithelial carcinoma (STIC) and adjacent normal epithelial cells. (A) Increased *LGR5* expression is observed in STICs with diffuse and strong P53 positivity, whereas adjacent normal epithelial cells also express significant amounts of *LGR5*. Representative images of markedly reduced *LGR5* expression in a STIC with strong P53-positivity (**B**) or no P53-positivity (**C**) compared to adjacent tubal epithelial cells. Red arrowheads indicate carcinoma cells and yellow arrowheads indicate adjacent normal tubal epithelium. (**D)** A table showing histo-scores of *LGR5* in a group of STIC and non-tumorous tubal epithelium (NTTE) (n =21). Yellow-colored numbers indicate higher scores between STIC and NTTE. Scale bar: 50 μm

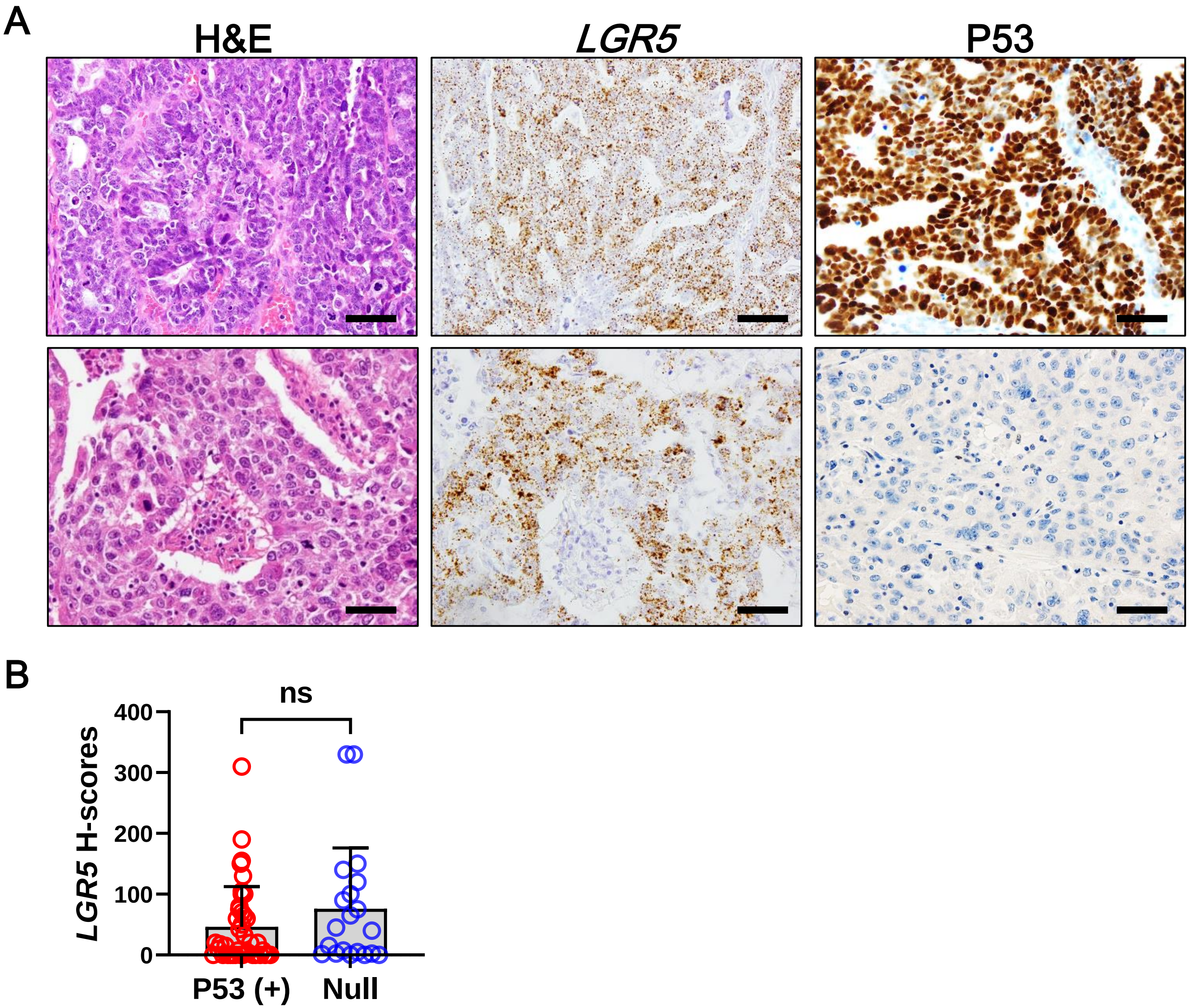

Supplementary Figure 5 Association of *LGR5* with P53 expression in high-grade serous carcinomas (HGSCs). (A) Representative images of HGSCs with high levels of *LGR5*. HGSCs display diffuse and strong positivity or complete negativity (null type) for P53. (**B)** A bar graph showing that there is no difference in *LGR5* histo-scores (H-scores) between P53-positive (n = 42) and null type (n = 20) HGSCs. Scale bar: 50 μm. The data are shown as the means ± SD. ns, not significant.

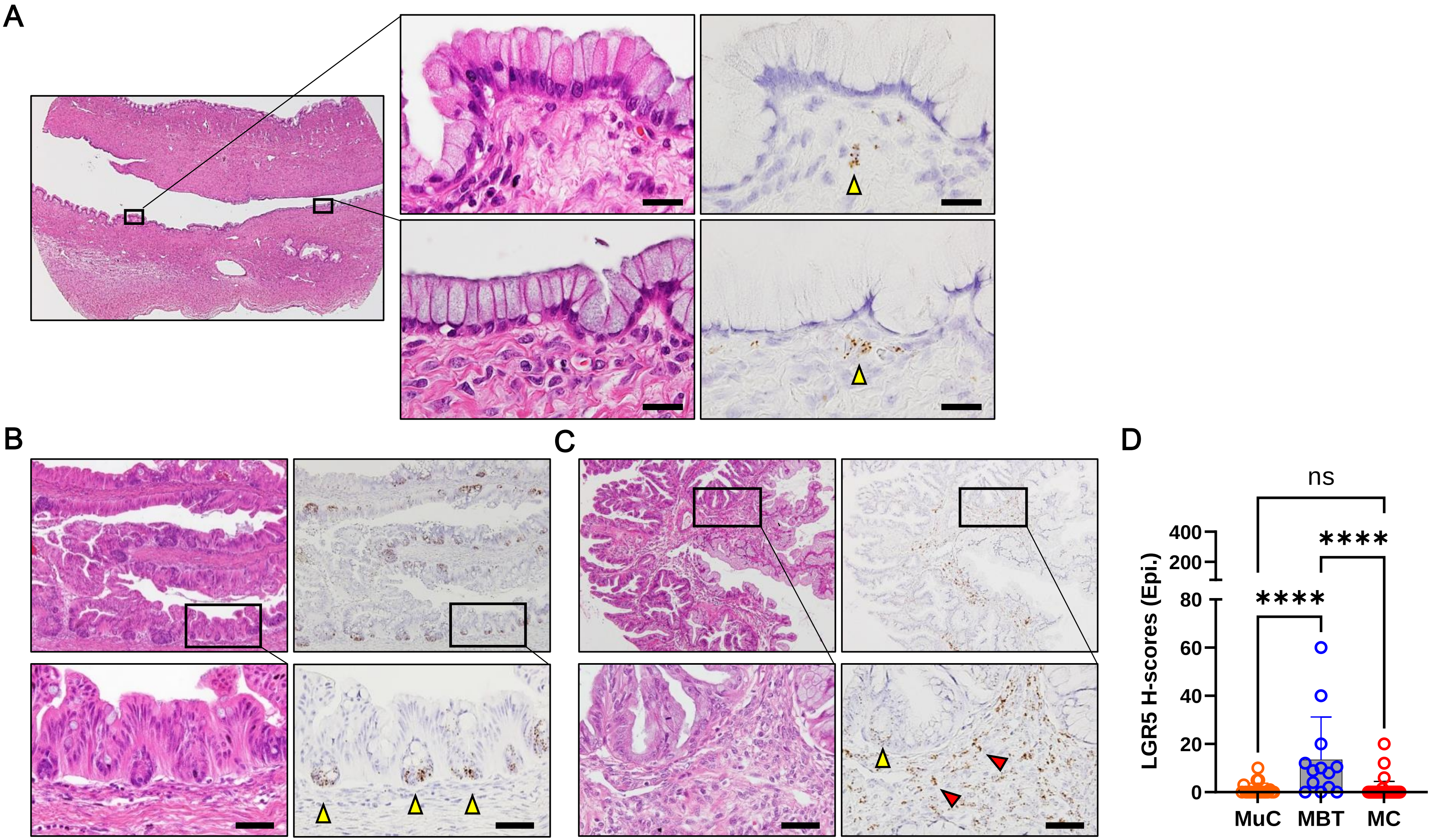

Supplementary Figure 6 ***LGR5*** **expression in mucinous cystadenoma and mucinous borderline tumor.** (**A**) *LGR5* expression is not observed in most mucinous epithelial cells (H-scores [mean±SD]: 1.1±2.6), but some subepithelial stromal cells (indicated by yellow arrowheads) are positive for *LGR5*. Scale bar, 20μm. (**B**) *LGR5* expression detected in borderline mucinous tumor is mostly confined to the basal areas of glands (H-scores [mean±SD]: 13.5±17.7). Scale bar, 20μm. **C** Some mucinous borderline tumors exhibit stronger positivity in stromal cells (indicated by red arrowheads) than in epithelial tumor cells. (indicated by yellow arrowhead). Scale bar, 50μm. **D** A bar graph showing *LGR5* H-scores in mucinous cystadenomas, mucinous borderline tumors, and mucinous carcinomas. The data are shown as the means ± SD. ns, not significant. ****P < 0.0001 by Tukey's multiple comparisons test.

Supplementary Figure 7 Original images of Western blot

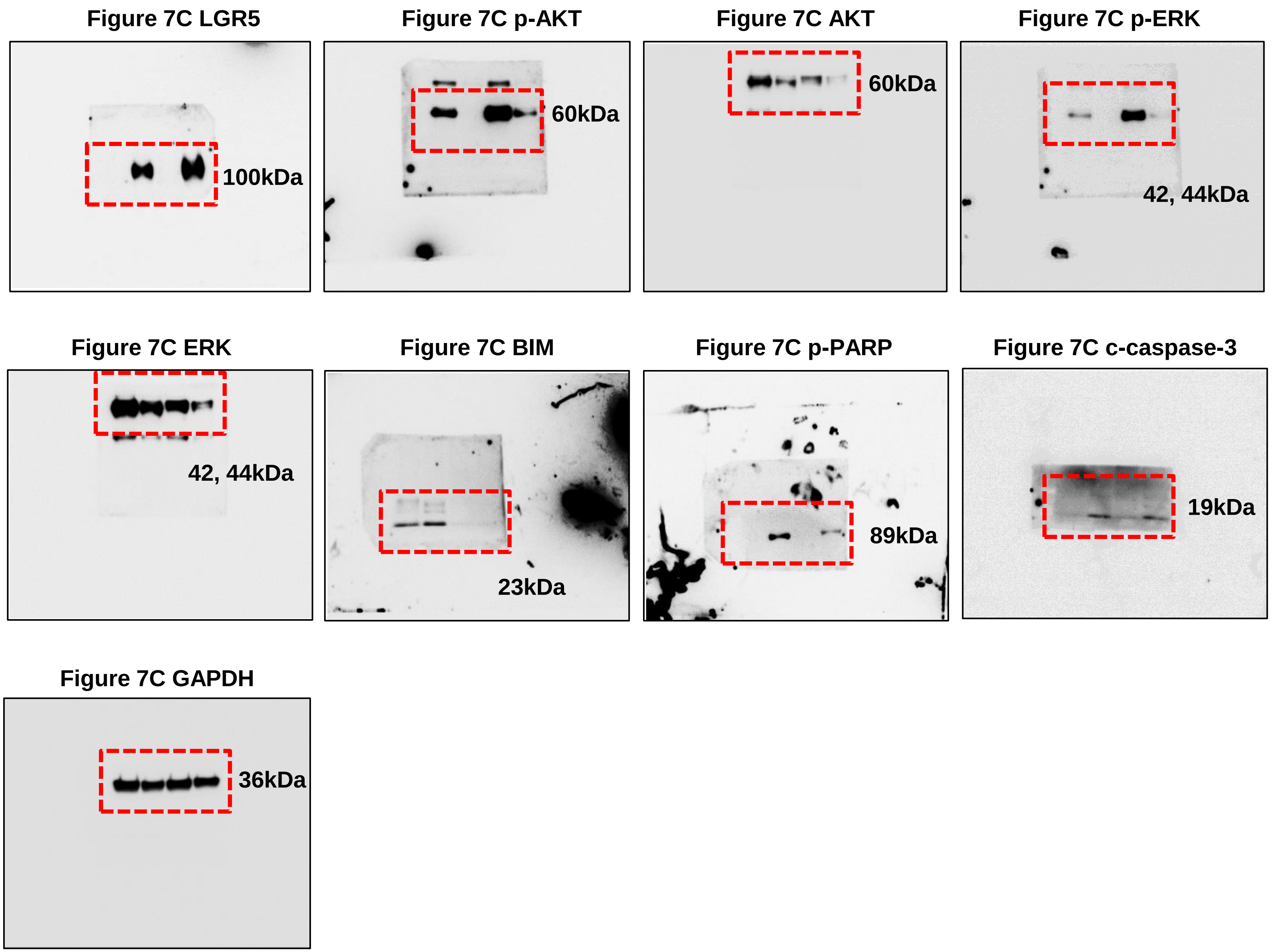

Supplement: Supplementary file 1 — Supplementary Information. [file 41598_2022_15234_MOESM1_ESM.pdf]
